# Hydrogen sulfide promotes lateral root formation in peach through persulfidation of SnRK1α kinase

**DOI:** 10.1111/pbi.70245

**Published:** 2025-07-04

**Authors:** Xuelian Wu, Anqi Du, Jiahui Liang, Zhe Wang, Yuansong Xiao, Futian Peng

**Affiliations:** ^1^ College of Horticulture Science and Engineering Shandong Agricultural University Taian China

**Keywords:** hydrogen sulfide, lateral root, PpSnRK1α, persulfidation, PpLBD16, *Prunus persica*

## Abstract

Root development is crucial for the growth and yield of horticultural crops. Hydrogen sulfide (H_2_S), an important gasotransmitter, has been shown to regulate lateral root (LR) formation in plants, including peach (*Prunus persica*). However, its specific regulatory mechanism remains largely unclear. Here, we show that the energy/metabolic sensor SUCROSE NON‐FERMENTING RELATED KINASE 1 (SnRK1) mediates the control of peach LR growth by H_2_S. PpSnRK1 activity in peach roots is enhanced with H_2_S to promote LR generation. Cys419, Cys430 and Cys505 residues in the catalytic α‐subunit of PpSnRK1 are modified by H_2_S persulfidation. Reduced persulfidation inhibits the H_2_S‐induced PpSnRK1 activity. Mutating Cys419 and Cys430 of PpSnRK1α severely impedes H_2_S‐promoted LR formation. LATERAL ORGAN BOUNDARIES DOMAIN 16 (LBD16) is a transcription factor essential for LR initiation. Further evidence shows that PpSnRK1α interacts with PpLBD16 in the nucleus, thereby enhancing the transcription of LR development‐related genes *PpCNGC1* (*CYCLIC NUCLEOTIDE‐GATED ION CHANNEL 1*) and *PpEXPB2* (*EXPANSIN‐B2*). In peach roots, transcription of these two genes is markedly up‐regulated by H_2_S‐induced PpSnRK1 activity. Silencing of *PpLBD16*, *PpEXPB2* or *PpCNGC1* significantly reduces exogenous H_2_S‐induced LR formation. In situ hybridization analysis shows that they are strongly expressed in peach LR primordia along with *PpSnRK1α*. Our data reveal an interaction between H_2_S signal and SnRK1 kinase, providing mechanistic insights into the shaping of agronomically important root systems.

## Introduction

The root system is essential for plants anchoring, absorbing water and nutrients, and adapting to the environment. LRs are the principal constituents of the root system, which are generated post‐embryonically from the primary root (PR) to increase the absorptive area for meeting plant growth requirements (Jia *et al*., 2022; Péret *et al*., 2009). The peach (*Prunus persica*), a fruit tree with shallow roots, is one of the most extensively cultivated fruit crops worldwide. The peach tree is a taproot system that has a distinct PR during the seedling stage, but as the seedling develops, the PR's growth slows down and the LR takes up most of the entire root system. Therefore, understanding the molecular regulatory mechanisms of LR development is helpful to improve the nutrient uptake and yield of peach.

Hydrogen sulfide (H_2_S), a gaseous signalling molecule that can cross biological membranes to transmit signals, is involved in the metabolism and development of plants and plays a positive role in plant environmental adaptation (Corpas and Palma, 2020; Zhang *et al*., 2021). It has been demonstrated that H_2_S regulates root organogenesis. Low concentrations of exogenous H_2_S stimulate root development in a variety of plants, such as sweet potato (*Ipomoea batatas*), tomato (*Solanum lycopersicum*), soybean (*Glycine max*) and *Arabidopsis* (*Arabidopsis thaliana*) (Fang *et al*., 2014; Jia *et al*., 2015; Zhang *et al*., 2009a). Through interacting with the auxin, hydrogen peroxide and nitric oxide pathways, the H_2_S signal promotes LR formation (Jia *et al*., 2015; Li *et al*., 2014; Mei *et al*., 2017). We also have earlier reported that 200 μM NaHS (sodium hydrogen sulfide, an exogenous H_2_S donor) has a positive effect on peach LR formation, which encourages LR growth by increasing the transcription of *LATERAL ORGAN BOUNDARIES DOMAIN 16* (*LBD16*) in the auxin pathway (Wu *et al*., 2021). Although these works reveal the role of H_2_S in LR formation, more research is needed to investigate the specific regulatory mechanisms.

Persulfidation, a reversible posttranslational modification of protein cysteine (Cys) residues that converts thiol groups (R‐SH) to persulfide groups (R‐SSH), is an important mechanism of H_2_S signalling (Filipovic *et al*., 2018; Gotor *et al*., 2019). Through persulfidation, H_2_S affects the structure, subcellular localisation, activity, and interactions of proteins in animals and plants (Aroca *et al*., 2017b; Chen *et al*., 2020; Paul and Snyder, 2015; Sun *et al*., 2021b). The persulfidation proteome reveals that 3147 proteins are persulfidated in the wild‐type *Arabidopsis*, and these proteins are involved in a wide range of biological processes, including carbon metabolism and plant development (Aroca *et al*., 2017a). Currently, it is known that the persulfidation modification regulates signalling pathways such as abscisic acid, ethylene and reactive oxygen species (ROS) (Jia *et al*., 2018; Shen *et al*., 2020; Zhou *et al*., 2021), modulating biological processes like flowering, autophagy and stomatal closure (Aroca *et al*., 2021; Laureano‐Marin *et al*., 2020; Ma *et al*., 2021; Shen *et al*., 2020). Root hair growth is also affected by actin persulfidation (Li *et al*., 2018). However, it is unclear whether persulfidation plays roles in H_2_S‐induced LR development.

LR formation requires carbon and energy inputs, which are closely linked to the plant energy regulatory network (Morales‐Herrera *et al*., 2024). The SUCROSE NON‐FERMENTING RELATED KINASE 1 (SnRK1) is responsible for the regulation of carbon/nitrogen metabolism and energy homeostasis, playing a critical regulatory role in plant growth (Nietzsche *et al*., 2018; Wang *et al*., 2022; Zacharaki *et al*., 2022). SnRK1 is a heterotrimeric complex composed of the catalytic α‐subunit and regulatory β and γ‐subunits (Emanuelle *et al*., 2016). The α‐subunit (SnRK1α) has catalytic activity independent of complexes, and its default activation has a key role for plant development (Ramon *et al*., 2019). Under normal conditions, SnRK1α is localised to the cytoplasm and nucleus, but low‐energy stress, low pH or high ammonium result in nuclear translocation of SnRK1α (Ramon *et al*., 2019; Sun *et al*., 2021a). In the nucleus, SnRK1α interacts with various transcription factors (TFs) to control downstream gene expression (Han *et al*., 2020b; Muralidhara *et al*., 2021; Wang *et al*., 2022). It has been reported that SnRK1α interacts with the BASIC LEUCINE ZIPPER 63 (bZIP63) to activate the *AUXIN RESPONSE FACTOR 19* (*ARF19*) in low‐energy conditions, thereby promoting *Arabidopsis* LR formation (Muralidhara *et al*., 2021). Interestingly, our previous investigations also found that PpSnRK1α can regulate peach LR development via the auxin pathway (Zhang *et al*., 2020).

SnRK1 is highly conserved with mammalian AMP‐ACTIVATED PROTEIN KINASE (AMPK) (Broeckx *et al*., 2016). In animals, H_2_S exerts cytoprotective or therapeutic functions through activating AMPK (Barr *et al*., 2015; Chen *et al*., 2016; Kundu *et al*., 2014; Wang *et al*., 2017; Xie *et al*., 2015). In plants, SnRK2.6 is activated by the persulfidation of H_2_S to enhance drought tolerance (Chen *et al*., 2020, 2021). SnRK1 belongs to the SnRK kinase family as SnRK2, but the relationship between H_2_S and SnRK1 is currently unknown.

In this work, we analysed the relationship between H_2_S and PpSnRK1 as well as the role of persulfidation in LR formation. We present evidence that H_2_S promotes LR formation through activation of PpSnRK1. We further show that the α‐subunit of PpSnRK1 is persulfidated by H_2_S. PpSnRK1α persulfidation mediates H_2_S‐induced LR development. We demonstrate that PpSnRK1α can interact with PpLBD16 to increase the transcription of downstream genes related to LR development. These findings provide molecular evidence for the interaction between the gasotransmitter H_2_S and the energy sensor SnRK1, revealing the H_2_S‐SnRK1α‐LBD16 regulation pathway and contributing to the modulation of peach root architecture in agricultural production.

## Results

### H_2_S enhances PpSnRK1 activity to induce LR formation

**Figure 1:**
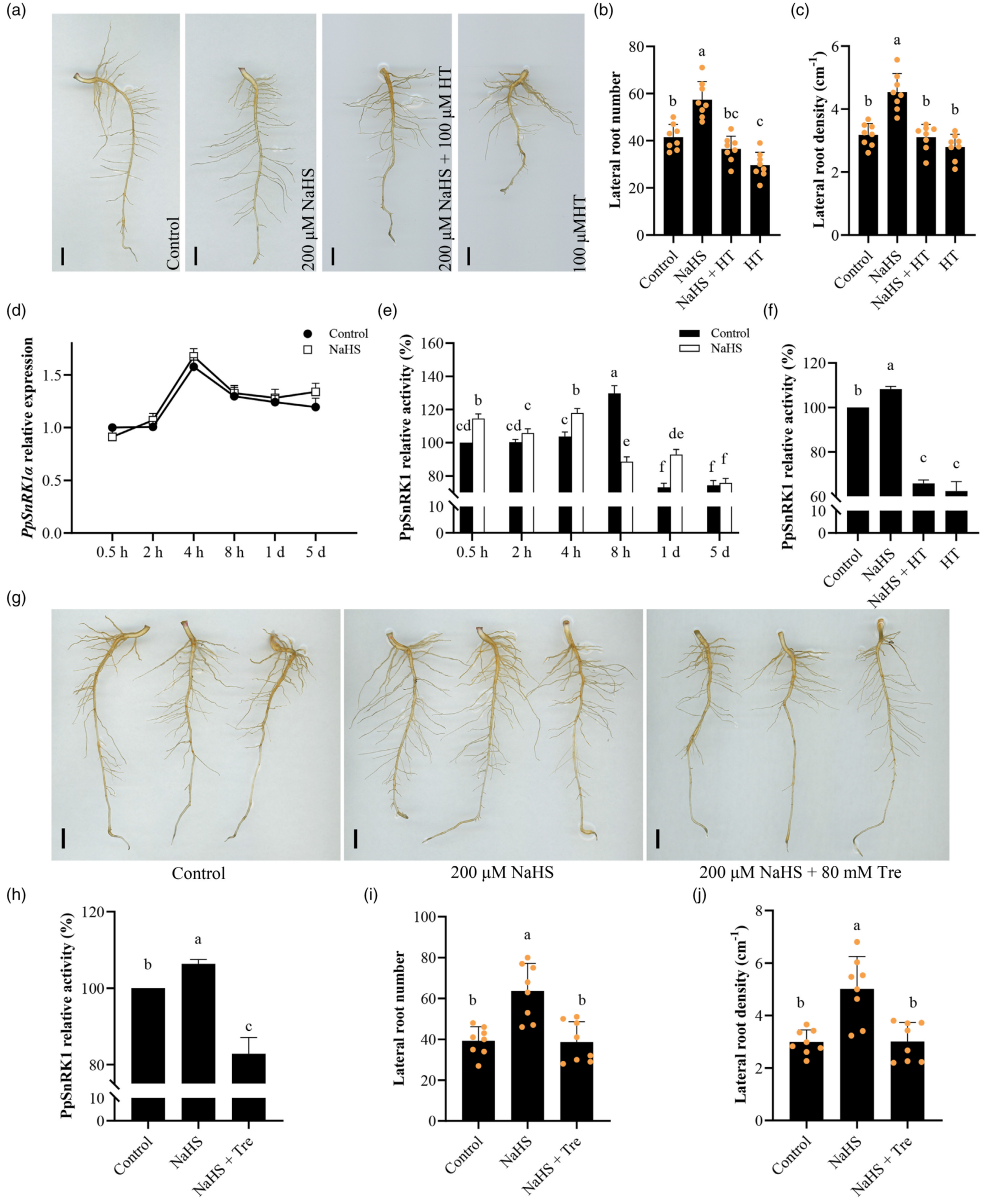
H_2_S induces LR formation by enhancing PpSnRK1 activity in peach roots. (a) Root phenotypes of peach seedlings treated for 5 days with water (control), 200 μM NaHS, 200 μM NaHS + 100μM HT and 100 μM HT, respectively. NaHS is an H_2_S donor, and HT is an H_2_S scavenger. Scale bars, 1 cm. (b and c) LR number (b) and density (c) of peach seedlings in the corresponding treatments in (a), *n* = 8. (d and e) Effect of NaHS (200 μM) on *PpSnRK1α* transcript levels (d) and PpSnRK1 activity (e) in peach roots at different time points after treatment, *n* = 3. (f) Effects of control, 200 μM NaHS + 100 μM HT and 100 μM HT treatments on PpSnRK1 activity in peach roots. On day 5 after the first treatment, another one was applied to the peach seedlings. The roots were sampled at 4 h after the 2nd treatment, *n* = 3. (g and h) Root phenotypes (g) and root PpSnRK1 activity (h) were analysed in control, 200 μM NaHS and 200 μM NaHS + 80 mM Tre‐treated peach seedlings. Root phenotypes were observed on day 5 after treatment. Scale bars, 1 cm. One further treatment was applied to peach seedlings on the 5th day following the initial one. Samples for the PpSnRK1 activity assay were taken at 4 h after the 2nd treatment, *n* = 3. (i and j) LR number (i) and density (j) of peach seedlings in the corresponding treatments in (g), *n* = 8. Data are mean with standard deviation (SD) of biological replicates (*n*). Statistical analyses were performed with one‐way ANOVA (b, c, f, h, i and j) or two‐way ANOVA (d and e). Different letters denote statistically significant differences (*p* < 0.05).

As we previously reported, H_2_S positively regulates peach LR development (Wu *et al*., 2021). The application of exogenous H_2_S (NaHS) obviously promoted the production of LR in peach seedlings, while the use of hypotaurine (HT), an H_2_S scavenger, blocked the induction of LR formation by H_2_S, and HT alone impeded root development compared to the control (Figure 1a–c). To understand whether PpSnRK1 is involved in this process, we detected the effects of NaHS treatment on the transcriptional level of the gene encoding the catalytic α‐subunit (PpSnRK1α) and the activity of PpSnRK1, respectively. We discovered that NaHS had minimal effect on PpSnRK1α transcript levels in peach roots compared to the control (Figure 1d). However, PpSnRK1 activity was rapidly enhanced with NaHS and remained in this higher active state for a period of time (0.5–4 h). On the 5th day of NaHS treatment, PpSnRK1 activity nearly returned to the control level (Figure 1e). We noticed that PpSnRK1 could be activated again by additional NaHS treatment on day 5. But this effect was eliminated by the application of HT. Using HT alone or combined with NaHS significantly suppressed PpSnRK1 activity (Figure 1f). These findings suggest that PpSnRK1 activity is positively regulated by H_2_S. To investigate whether the PpSnRK1 activity mediated H_2_S regulation of LR development, we treated peach seedlings with trehalose (Tre), an inhibitor for SnRK1 activity (Figure 1g,h). Root phenotypes of peach showed that the use of Tre inhibited H_2_S‐induced LR development, reducing both the number and density of LRs down to control levels (Figure 1i,j). These results indicate that H_2_S promotes peach LR formation by activating PpSnRK1 kinase.

### Cys419, Cys430 and Cys505 residues in the catalytic α‐subunit of PpSnRK1 are persulfidated by H_2_S

**Figure 2:**
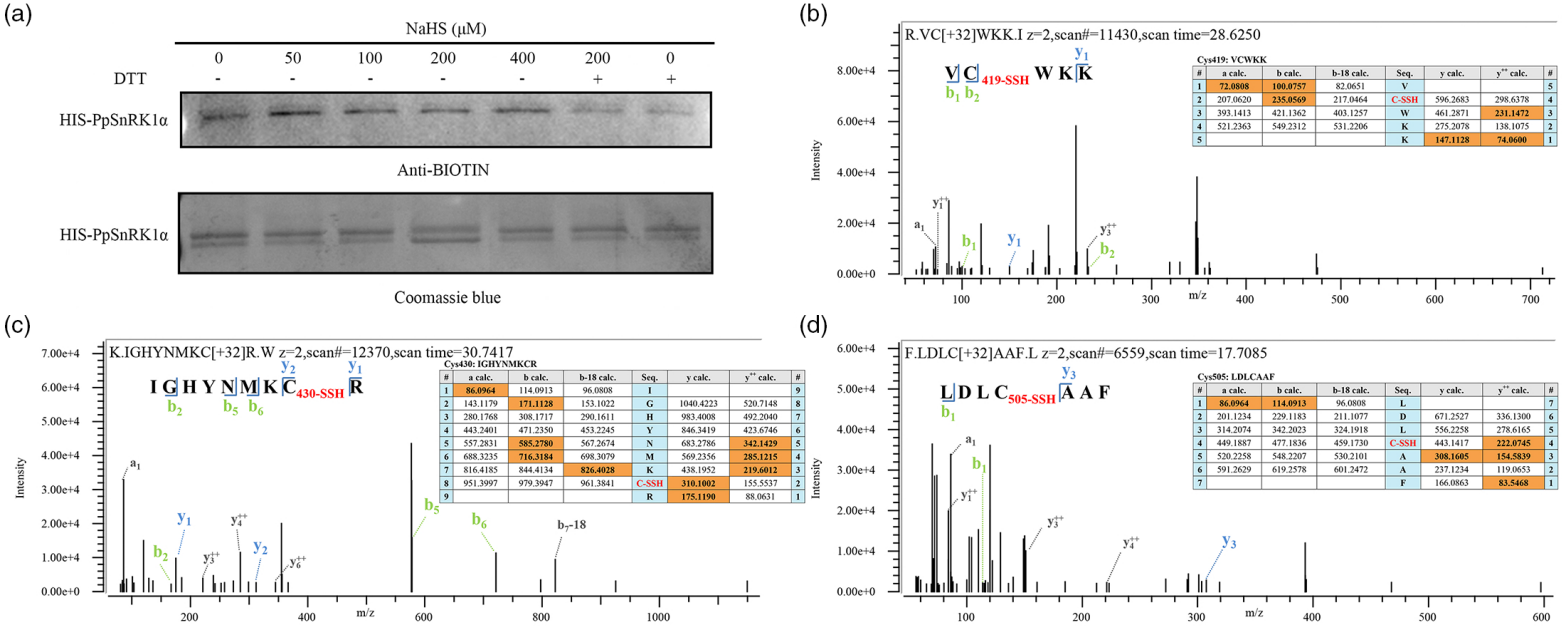
C419, C430 and C505 residues of PpSnRK1α are modified by persulfidation. (a) Persulfidation analysis of recombinant PpSnRK1α *in vitro*. The same concentration of HIS‐PpSnRK1α recombinant protein was incubated with a gradient concentration (0–400 μM) of NaHS for 30 min in the presence or absence of 10 mM DTT. Persulfidated PpSnRK1α was detected by immunoblot analysis using anti‐biotin antibodies. (b–d) LC–MS/MS analysis of persulfidated sites of recombinant PpSnRK1α. The peptides of PpSnRK1α containing C419 (b), C430 (c) and C505 (d) were detected to be persulfidated. The predicted ion types and molecular masses of the modified peptides are shown in the table inside the mass spectrum. The ions detected in the spectrum are highlighted in orange, while the persulfidated amino acids are indicated in red.

By persulfidation, H_2_S can alter the activity of target proteins (Chen *et al*., 2020; Laureano‐Marin *et al*., 2020). To analyse whether PpSnRK1 is affected by this modification, we performed a persulfidation assay of recombinant PpSnRK1α *in vitro* and detected it by immunoblotting using anti‐biotin antibodies. The results showed that the biotin‐labelled persulfidated PpSnRK1α was clearly detected with the antibody, particularly under NaHS treatment. In contrast, the use of dithiothreitol (DTT) greatly reduced the level of biotin‐labelled PpSnRK1α (Figure 2a), implying that PpSnRK1α is a target protein for persulfidation. In order to identify the persulfidation site of PpSnRK1α, we performed liquid chromatography–tandem mass spectrometry (LC–MS/MS) analysis of the recombinant PpSnRK1α treated with NaHS. The proteins were digested using trypsin or chymotrypsin, and the obtained peptides were analysed by mass spectrometry. There were three identified peptides with a molecular mass increase of 32 Da, including VCWKK, IGHYNMKCR and LDLCAAF. The mass spectrum showed that Cys419 (C419) in VCWKK, Cys430 (C430) in IGHYNMKCR and Cys505 (C505) in LDLCAAF were modified by persulfidation (Figure 2b–d).

### H_2_S induces PpSnRK1 activity based on persulfidation

**Figure 3:**
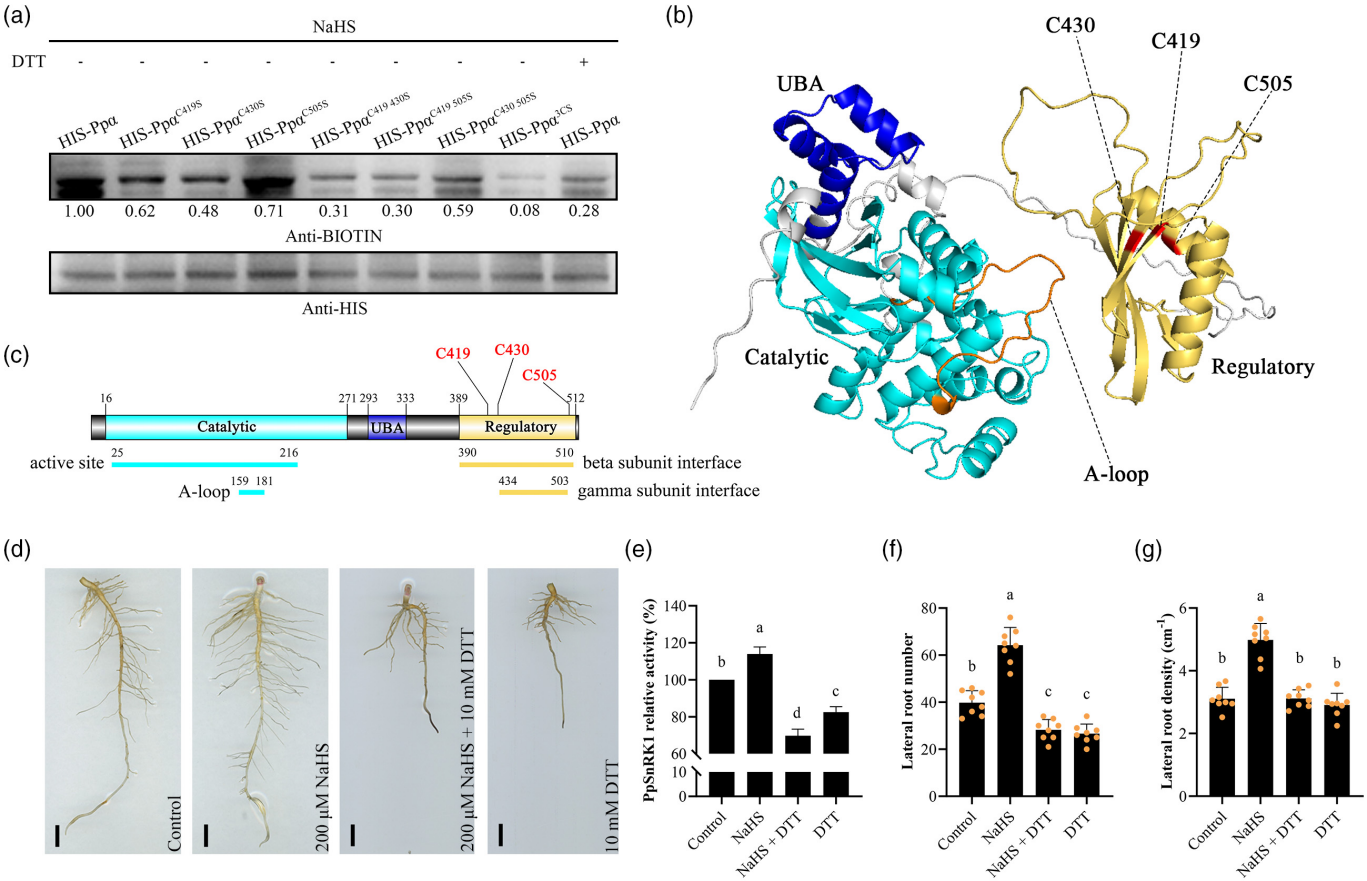
H_2_S persulfidates PpSnRK1α to enhance the activity of PpSnRK1. (a) *In vitro* persulfidation analysis of HIS‐PpSnRK1α (HIS‐Ppα) for C419, C430 and C505 mutations under NaHS treatment. Cys (C) was mutated to Ser (S), and 3CS represents the C419 430 505S three‐site mutation. The recombinant proteins were incubated with 200 μM NaHS in the presence or absence of 10 mM DTT for 30 min. By utilising anti‐biotin antibodies in immunoblot analysis, persulfidated PpSnRK1α was identified. ImageJ software was used to quantify the abundance of proteins. The ratio of protein detected by anti‐BIOTIN to anti‐HIS was used to assess the level of persulfidated PpSnRK1α. The numbers below the lanes indicate the relative persulfidation levels of the mutants compared to the wild‐type HIS‐PpSnRK1α persulfidation level (1.00). Similar results were observed in three independent experiments. (b and c) Predicted structural model (b) and core domain distribution (c) of PpSnRK1α. A‐loop represents the activation loop. UBA represents the ubiquitin‐associated domain. The numbers indicate amino acid positions. The colour of the domains in (c) corresponds to that of the domains in (b). PpSnRK1α persulfidation sites C419, C430 and C505 are marked in red. (d and e) Root phenotypes (d) and root PpSnRK1 activity (e) were analysed in control, 200 μM NaHS, 200 μM NaHS + 10 mM DTT and 10 mM DTT‐treated peach seedlings. Root phenotypes were observed on day 5 after treatment. Scale bars, 1 cm. One further treatment was applied to peach seedlings on the 5th day following the initial one. Samples for the PpSnRK1 activity assay were taken at 4 h after the 2nd treatment, *n* = 3. (f and g) LR number (f) and density (g) of peach seedlings in the corresponding treatments in (d), *n* = 8. Data are mean with SD of biological replicates (*n*). Statistical analyses were performed with one‐way ANOVA (d, f and g). Different letters denote statistically significant differences (*p* < 0.05).

To further determine residues C419, C430 and C505 as the persulfidation sites of PpSnRK1α, by mutating Cys to Ser (S), we constructed persulfidation site single/double/triple mutant PpSnRK1α, purified the mutant recombinant proteins and assessed the impact of H_2_S on the persulfidation modification of them (Figure 3a). Immunoblotting using anti‐biotin antibodies showed that single‐site mutations PpSnRK1α^C419^, PpSnRK1α^C430^ and PpSnRK1α^C505^ resulted in decreased levels of persulfidation under NaHS treatment, especially PpSnRK1α^C419S^ and PpSnRK1α^C430S^. Random two‐site mutations further impeded H_2_S‐induced persulfidation. The persulfidation level of PpSnRK1α^C419 430S^ and PpSnRK1α^C419 505S^ was similar to that of PpSnRK1α treated with DTT. PpSnRK1α^3CS^, a three‐site mutation, almost completely eliminated H_2_S‐induced persulfidation. These findings demonstrate that C419, C430 and C505 are the persulfidation sites of PpSnRK1α.

Using the soybean (*Glycine max*) SnRK1α (alias GmKIN10) structure as a template, we utilised homology modelling to predict the three‐dimensional structure of PpSnRK1α in order to understand the location of the persulfidation sites in the structure (Figure 3b). Peach‐PpSnRK1α, soybean‐GmKIN10 and *Arabidopsis*‐AtSnRK1.1 protein sequences are homologous. The activation loop (A‐loop) as well as the three persulfidated sites, C419, C430 and C505, are highly conserved in these sequences (Figure S1). The predicted structure of PpSnRK1α shows that all of the persulfidation sites are located in the regulatory domain (yellow region, Figure 3b,c), which is responsible for interacting with the regulatory β‐subunit. These locations are spatially close to the A‐loop (orange region, Figure 3b), implying that the persulfidation sites might have an impact on PpSnRK1 activity.

Covalent modification of persulfidation can be reduced by DTT (Chen *et al*., 2021; Laureano‐Marin *et al*., 2020) (Figures 2a and 3a). We applied 10 mM DTT to peach seedlings to examine the impact of persulfidation on PpSnRK1 activity (Figure 3d). Compared to the control, the DTT treatment alone or in combination with NaHS greatly reduced the activity of PpSnRK1 (Figure 3e), severely impeding the peach root's development (Figure 3d). Compared with NaHS treatment, the use of DTT led to a considerable decrease in the number and density of LRs (Figure 3f,g). Together with the above results, we suggest that H_2_S can control the development of peach roots by increasing PpSnRK1 activity through persulfidation.

### Persulfidation sites Cys419 and Cys430 of PpSnRK1α mediate H_2_S‐induced LR formation

**Figure 4:**
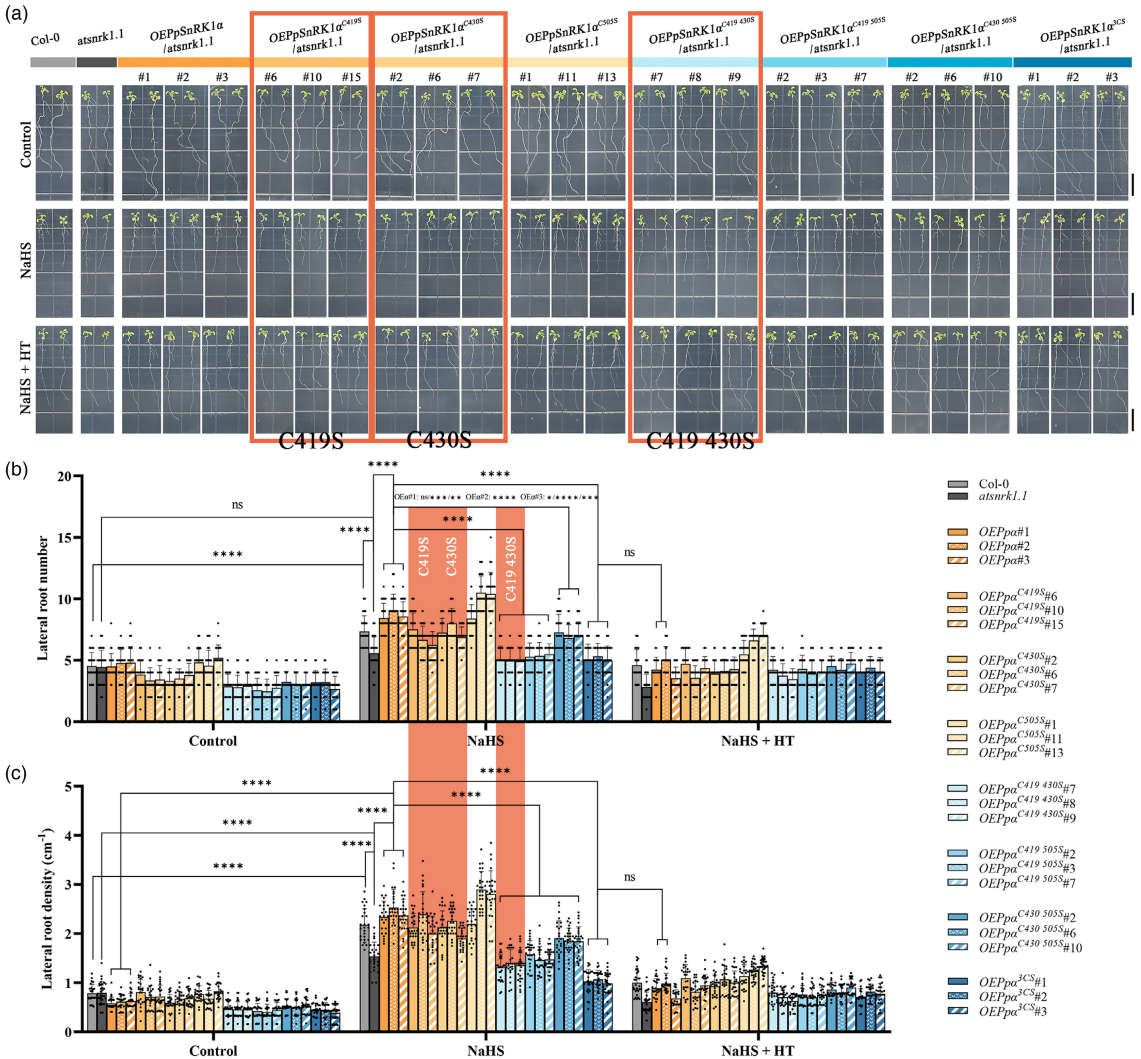
C419 and C430 residues are critical for H_2_S‐PpSnRK1α pathway‐induced LR formation. (a) *Arabidopsis* root phenotypes of 9‐day‐old Col‐0, *atsnrk1.1*, *OEPpSnRK1α/atsnrk1.1* and different persulfidation site mutants *OEPpSnRK1α* ^*C419/430/505S*^ */atsnrk1.1*. Seedlings were grown on half‐strength Murashige and Skoog (0.5x MS) medium with or without 200 μM NaHS or 100 μM HT. Scale bars, 1.5 cm. (b and c) LR number (b) and density (c) of *Arabidopsis* in (a), *n* ≥ 25. *Ppα* is an abbreviation for *PpSnRK1α*. The phenotypes and data for the key persulfidation site mutants are highlighted and labelled. Data are mean with SD of biological replicates (*n*). Statistical analyses were performed with two‐way ANOVA, ****p < 0.0001; ***p < 0.001; **p < 0.01; *p < 0.05; ns = not significant.

Due to the lack of a stable genetic transformation system in peach, we cannot obtain a *ppsnrk1α* mutant for functional validation. To demonstrate the involvement of the PpSnRK1α persulfidation sites in H_2_S‐induced LR formation, we heterologously overexpressed *PpSnRK1α* (*OEPpSnRK1α*) or the single/double/triple persulfidation site‐mutated *PpSnRK1α* (*OEPpSnRK1α* ^*C419/430/505S*^) in the *atsnrk1.1* background (Figure 4a). As compared to Col‐0, the *atsnrk1.1* mutation significantly reduced the number and density of LRs under NaHS treatment, lowering root sensitivity to H_2_S. This suggests that AtSnRK1.1 responds to exogenous H_2_S and has a role in LR formation. Under control or NaHS with additional HT, there were comparatively small changes in LR number/density between the lines of *atsnrk1.1*, *OEPpSnRK1α* and *OEPpSnRK1α* ^*C419/430/505S*^. But when these lines were treated with NaHS, there was a noticeable increase in the difference in LR number/density between them (Figure 4b,c). These results indicate that PpSnRK1α and its persulfidation sites are involved in LR occurrence in response to exogenous H_2_S.

Under NaHS treatment, overexpressing *PpSnRK1α* raised the sensitivity of the *atsnrk1.1* mutant to H_2_S, which led to a significant increase in both LR number and density. In comparison to the *OEPpSnRK1α* transgenic lines, the *PpSnRK1α* ^*C419S*^ or *PpSnRK1α* ^*C430S*^ single‐site mutations suppressed the emergence of LR caused by NaHS. This inhibitory effect was strengthened by the double‐site mutations *PpSnRK1α* ^*C419 430S*^ or *PpSnRK1α* ^*C419 505S*^, which decreased the LR number and density to the atsnrk1.1 level. Compared to *OEPpSnRK1α* ^*C419 430S*^ and *OEPpSnRK1α* ^*C419 505S*^ lines, the LR number of *OEPpSnRK1α* ^*3CS*^ didn't reduce further in NaHS treatment, and its LR number and density were comparable to those of the *OEPpSnRK1α* lines treated with HT added to NaHS. Notably, the *PpSnRK1α* ^*C505S*^ mutation didn't hinder H_2_S‐induced LR generation compared to *OEPpSnRK1α*. With NaHS treatment, the *PpSnRK1α* ^*C430 505S*^ two‐site mutation also had a weaker inhibitory effect on LR number/density than the other two‐site mutations (Figure 4b,c). In conclusion, mutations in residues C419 and C430 almost entirely prevented PpSnRK1α from mediating H_2_S‐induced LR formation. These phenotypes show that the persulfidation sites C419 and C430 play important roles in H_2_S‐PpSnRK1α‐induced LR formation.

Moreover, under control, we found that all of the persulfidation site mutant lines, except C505S, showed a decreasing trend in LR number compared to the *OEPpSnRK1α* lines, but no distinct trend was observed in the presence of HT (Figure 4b,c), implying that PpSnRK1α also controls LR development in response to endogenous H_2_S.

**Figure 5:**
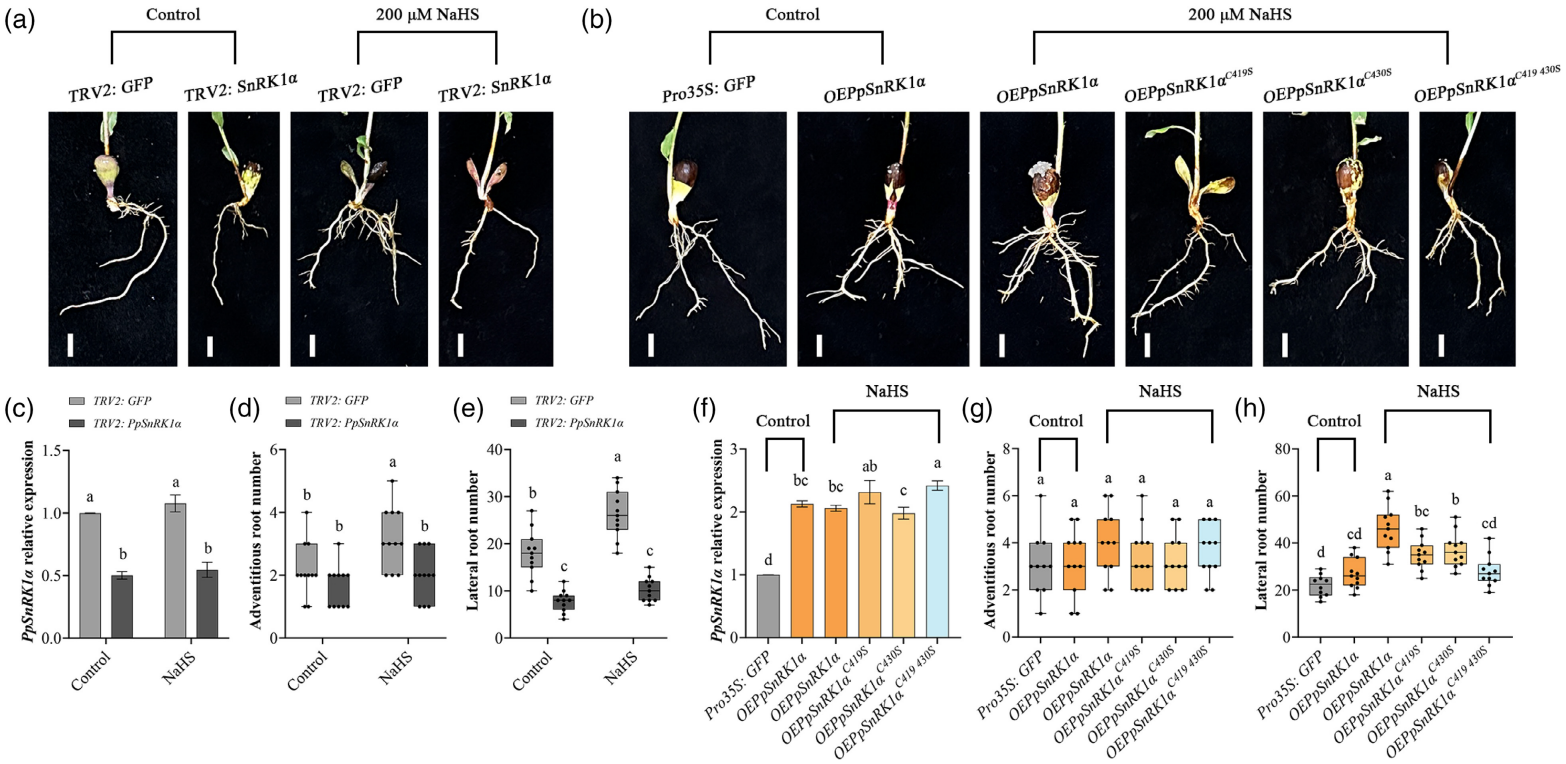
PpSnRK1α and its key persulfidation sites C419 and C430 are involved in exogenous H_2_S‐induced peach LR formation. (a) Peach root phenotypes of *PpSnRK1α*‐silenced line treated with 200 μM NaHS for 5 days. Scale bars, 1 cm. (b) Peach root phenotypes of *PpSnRK1α* overexpressing line or *PpSnRK1α* overexpressing lines with C419 or/and C430 mutations treated for 5 days with 200 μM NaHS. Scale bars, 1 cm. (c) Relative expression levels of *PpSnRK1α* in the peach roots shown in (a), *n* = 3. (d and e) Number of adventitious roots (d) and LRs (e) of peach seedlings in (a), *n* ≥ 10. (f) Relative expression levels of *PpSnRK1α* in the peach roots shown in (b), *n* = 3. (g and h) Number of adventitious roots (g) and LRs (h) of peach seedlings in (b), *n* ≥ 10. Peach roots were treated after 21 days of transformation. In column charts, data are mean with SD of biological replicates (*n*). In boxplots, centre line = median; whiskers = highest and lowest values; box limits = upper and lower quartiles; points = single measures. Statistical analyses were performed with two‐way ANOVA (c–e) or one‐way ANOVA (f, g and h). Different letters denote statistically significant differences (*p* < 0.05).

To ascertain whether PpSnRK1α and its key persulfidation sites, C419 and C430, play the same role in peach root development, we further analysed the effect of H_2_S on peach root phenotypes of *PpSnRK1α*‐silenced or overexpressed lines using transient transformation technology (Figure 5a,b). Under the control treatment, we observed that the number of adventitious roots produced by the VIGS (virus‐induced gene silencing)‐mediated *PpSnRK1α*‐silenced plants showed no significant change compared with that of the empty *TRV2* line, but the number of LRs formed on adventitious roots was significantly reduced (Figure 5a,c–e). Under NaHS treatment, the number of adventitious roots and LRs was significantly increased in the *TRV2* line, but silencing *PpSnRK1α* inhibited the induction by NaHS (Figure 5d,e), suggesting that PpSnRK1α can mediate exogenous H_2_S to regulate peach LR development. Similarly, *PpSnRK1α* overexpression had no significant effect on adventitious root formation (Figure 5f,g). A notable rise in LR number on adventitious roots of *OEPpSnRK1α* line was caused by NaHS (Figure 5b,h). However, single or double mutations at the persulfidation sites C419 and C430 significantly decreased LR number compared to the *OEPpSnRK1α* line under NaHS treatment (Figure 5b,h), suppressing the induction of LR formation by NaHS. These findings reaffirm that exogenous H_2_S induces LR production through the mediation of PpSnRK1α and its persulfidation sites C419 and C430.

### PpSnRK1α interacts with the transcription factor PpLBD16

Our earlier research discovered that PpSnRK1α has interactions with the auxin pathway to regulate peach root development (Zhang *et al*., 2020). *LBD16* is a core TF in the auxin pathway promoting LR initiation (Goh *et al*., 2019; Lee *et al*., 2009). In a previous transcriptome, we found that *PpLBD16* can respond to exogenous H_2_S to induce peach LR formation (Wu *et al*., 2021). Therefore, we speculated that PpSnRK1α may regulate peach LR formation via PpLBD16.

**Figure 6:**
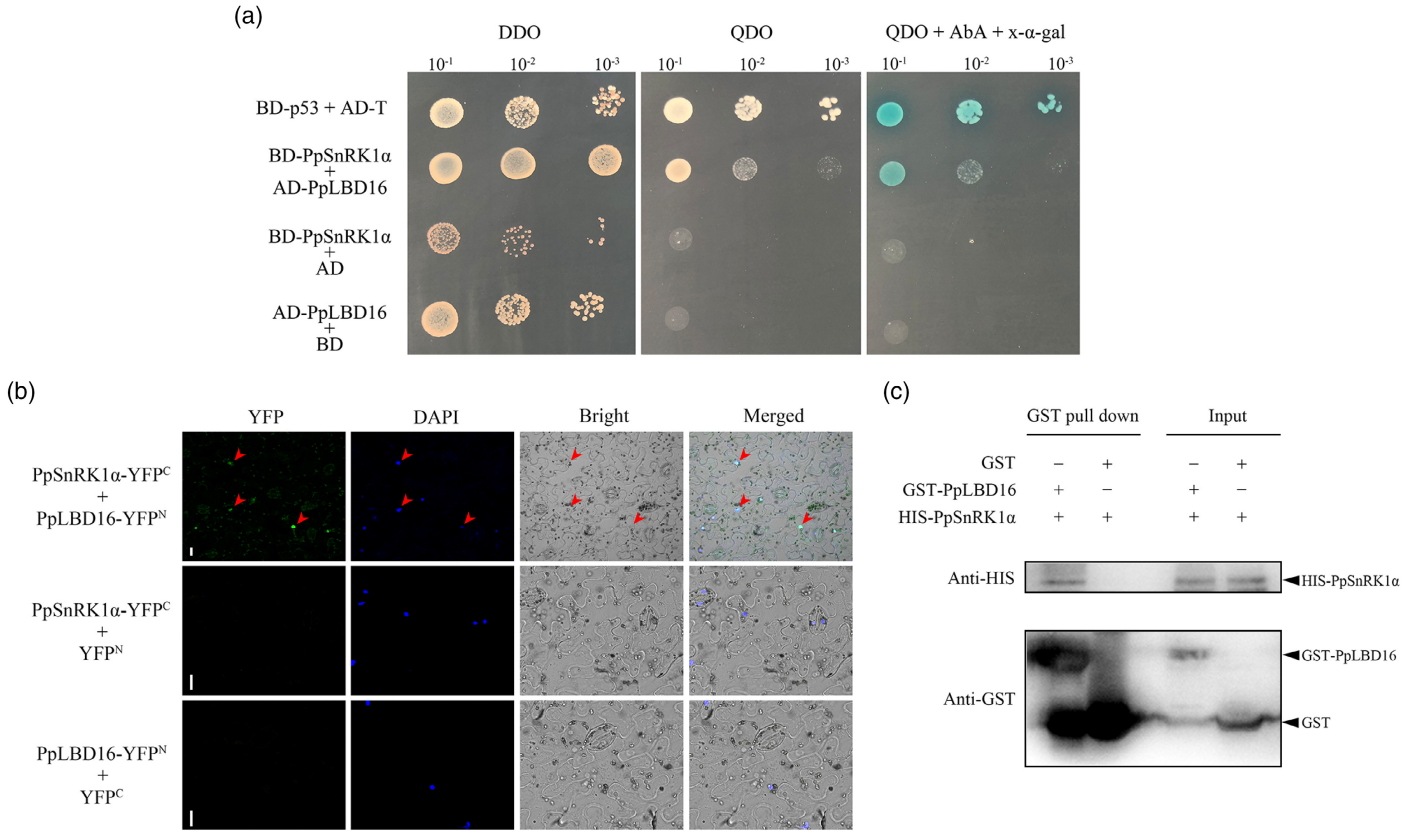
PpSnRK1α interacts with PpLBD16. (a) Yeast two‐hybrid assay of PpSnRK1α with PpLBD16. BD, pGBKT7; AD, pGADT7. BD‐p53 in combination with AD‐T is a positive control, and combinations containing empty BD or AD are negative controls. AbA was utilised at a 100 ng/mL concentration, while the X‐α‐gal concentration was 100 μg/mL. DDO, SD/‐Trp/‐Leu; QDO, SD/‐Ade/‐His/‐Trp/‐Leu. (b) BIFC analysis showing the interaction of PpSnRK1α with PpLBD16 in the nucleus of *N. benthamiana* leaf cells. The YFP system was used for detection. YFP^C^ (pSPYCE) and YFP^N^ (pSPYNE) indicate empty vectors and served as the controls. DAPI (4′, 6‐diamidino‐2‐phenylindole) was used as a nuclear dye. Red arrows point to the location of the interaction. Scale bars, 20 μm. (c) GST pull‐down assay between recombinant HIS‐PpSnRK1α and recombinant GST‐PpLBD16. The combination of GST and HIS‐PpSnRK1α was used as a control. In 10% (w/v) sodium dodecyl sulfate‐polyacrylamide gel electrophoresis (SDS‐PAGE), 20 μL of samples were loaded for GST pull‐down and 10 μL of samples were loaded for input detection. The recombinant proteins were detected by immunoblot analysis using anti‐HIS and anti‐GST antibodies, respectively. Two independent experiments showed similar results.

To verify this hypothesis, we performed protein–protein interaction analyses. We carried out a yeast two‐hybrid assay using pGBK7‐PpSnRK1α (BD‐PpSnRK1α) as the bait and pGADT7‐PpLBD16 (AD‐PpLBD16) as the prey. As shown in Figure 6a, AD‐PpLBD16 and BD‐PpSnRK1α cotransformed yeast were able to grow on quadruple selection medium SD/‐Ade/‐His/‐Trp/‐Leu (QDO). Colonies coloured blue when X‐α‐gal (5‐bromo‐4‐chloro‐3‐indoxyl‐α‐D‐galactopyranoside) was added, which was consistent with the positive control. In contrast, yeast cotransformed with the empty vector AD or BD (negative control) did not grow on QDO (Figure 6a). These suggest that there is an interaction between PpSnRK1α and PpLBD16. Bimolecular fluorescence complementation (BIFC) assays further confirmed that PpSnRK1α interacted with PpLBD16 in the nucleus of *Nicotiana benthamiana* leaf cells (Figure 6b). Similarly, the *in vitro* pull‐down assay also detected that the recombinant HIS‐PpSnRK1α could be enriched by the recombinant GST‐PpLBD16, while control GST could not (Figure 6c). The findings above indicate that PpSnRK1α can directly interact with PpLBD16.

In addition, we analysed the effect of exogenous H₂S on the subcellular localisation of PpSnRK1α. By observing the subcellular localisation of the PpSnRK1α‐GFP fusion protein in the root apical meristems of *OEPpSnRK1α/atsnrk1.1 Arabidopsis* seedlings, we found that PpSnRK1α‐GFP was localised in the cytoplasm and nucleus (Figure S2a). Interestingly, as compared to the control, the ratio of the mean nuclear and mean cytoplasmic fluorescence intensity (N/C) of the PpSnRK1α‐GFP was significantly increased under exogenous H_2_S treatment (Figure S2a,b), suggesting that exogenous H_2_S enhances the nuclear localisation of PpSnRK1α. However, when mutated persulfidation sites C419 and C430, the N/C ratio of the PpSnRK1α^C419 430S^‐GFP fusion protein was nearly decreased to the control level (Figure S2a,b), indicating that the persulfidation of H_2_S promotes the nuclear localisation of PpSnRK1α. This improvement in PpSnRK1α's nuclear localisation might make it easier for PpSnRK1α and PpLBD16 to interact.

### H_2_S enhances transcriptional activation of target genes by PpLBD16 via PpSnRK1α

**Figure 7:**
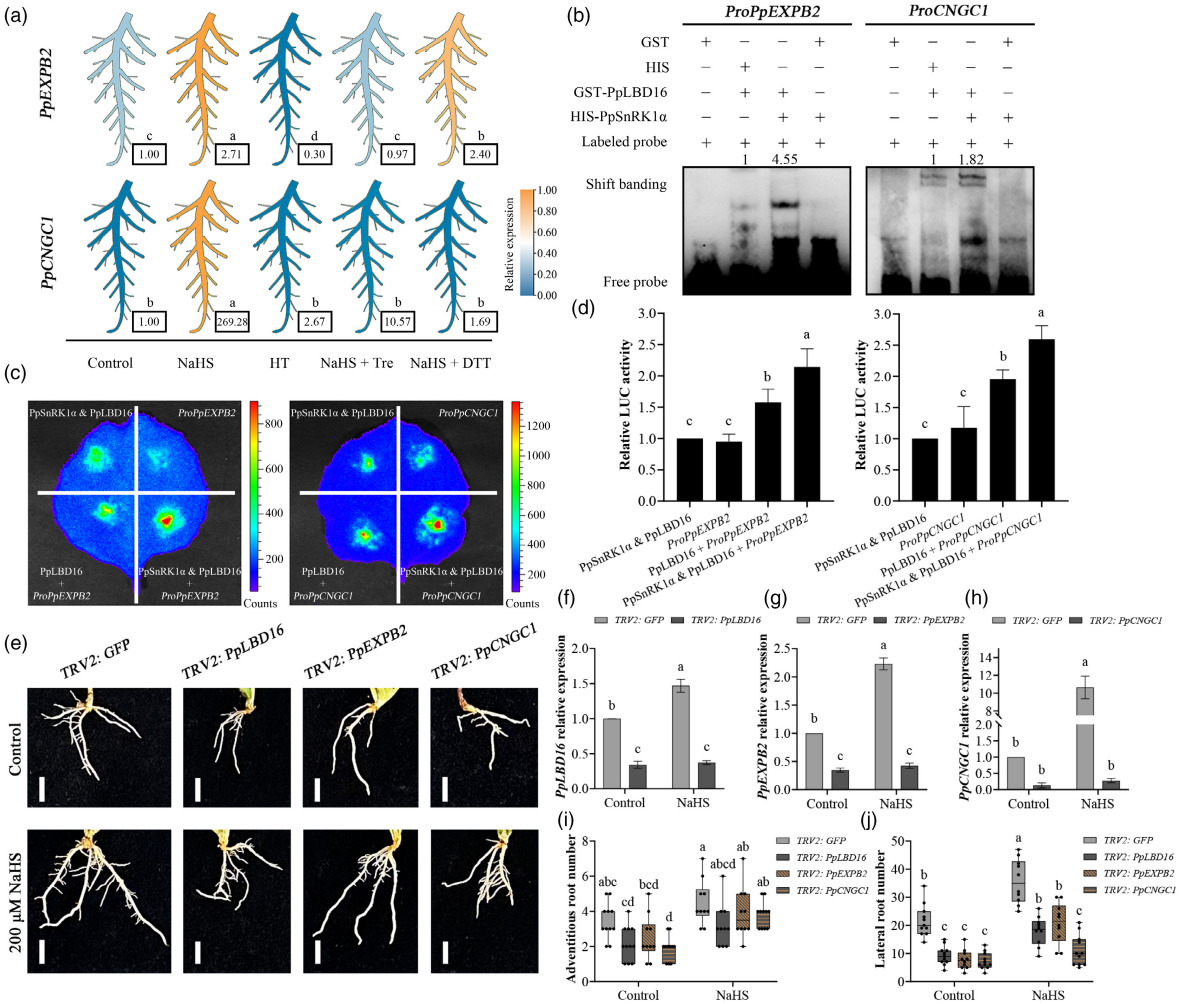
H_2_S promotes the transcription of downstream genes related to LR development via PpSnRK1α‐PpLBD16. (a) *PpEXPB2* and *PpCNGC1* relative expression in the roots of control, 200 μM NaHS, 100 μM HT, 200 μM NaHS + 80 mM Tre and 200 μM NaHS + 10 mM DTT‐treated peach seedlings was determined by RT‐qPCR. One further treatment was applied to peach seedlings on the 5th day following the initial one. Samples for RT‐qPCR analysis were taken at 4 h after the 2nd treatment. The relative expression of the heatmap was standardised by row Z‐score. Unstandardised data shown in black boxes are means of three biological replicates. (b) EMSA analysis of PpSnRK1α's effect on PpLBD16 binding to the promoters of *PpEXPB2* and *PpCNGC1*. Probes for the promoters were labelled with biotin. Using ImageJ software, the relative intensity of the binding bands was quantified and marked at the top of the lane. Similar results were observed in three independent experiments. (c) Dual‐luciferase transient expression assay for the effect of PpSnRK1α cotransfection with PpLBD16 on the transcription of *PpEXPB2* and *PpCNGC1*. This analysis was performed by infesting leaves of *N. benthamiana* according to different combinations. (d) Relative LUC activity of *PpEXPB2* and *PpCNGC1* in (c), *n* = 3. Relative activity was calculated for each group based on the comparison with the level of the ‘PpSnRK1α & PpLBD16’ group (1.00). (e) Peach root phenotypes of *PpLBD16*, *PpEXPB2* or *PpCNGC1*‐silenced line treated with 200 μM NaHS for 5 days. Scale bars, 1 cm. (f‐h) Relative expression levels of *PpLBD16* (f), *PpEXPB2* (g) or *PpCNGC1* (h) in peach roots of the silencing lines shown in (e), *n* = 3. (i and j) Number of adventitious roots (i) and LRs (j) of peach seedlings in (e), *n* ≥ 10. Peach roots were treated after 23 days of transformation. In column charts, data are mean with SD of biological replicates (*n*). In boxplots, centre line = median; whiskers = highest and lowest values; box limits = upper and lower quartiles; points = single measures. Statistical analyses were performed with one‐way ANOVA (a and d) or two‐way ANOVA (f–j). Different letters denote statistically significant differences (*p* < 0.05).

Our prior research revealed that *EXPANSIN‐B2* (*PpEXPB2*), *CYCLIC NUCLEOTIDE‐GATED ION CHANNEL 1* (*PpCNGC1*), *SUBTILISIN‐LIKE PROTEASE 1.7* (*PpSBT1.7*) and *POLYPHENOL OXIDASE* (*PpPPO*) are the target genes of PpLBD16 to promote LR formation (Wu *et al*., 2024). To investigate whether these target genes' transcription is affected by the H_2_S‐PpSnRK1α pathway, we conducted reverse transcription quantitative PCR (RT‐qPCR) analysis on peach roots treated with NaHS, HT, NaHS + Tre and NaHS + DTT. Compared to the control, *PpSBT1.7* and *PpPPO* expression altered minimally under NaHS or NaHS + Tre treatment (Figure S3). However, the transcription of *PpEXPB2* and *PpCNGC1* was significantly up‐regulated by NaHS, of which *PpCNGC1* was strongly expressed (Figure 7a). By employing HT to scavenge endogenous H_2_S, *PpEXPB2* expression was significantly down‐regulated, showing that it is also influenced by endogenous H_2_S. When Tre or DTT was applied to block NaHS‐induced PpSnRK1 activity (Figures 1h and 3e), *PpCNGC1* transcription was decreased dramatically or dropped to the control level. The induction effect of NaHS on *PpEXPB2* expression was also weakened by DTT or significantly blocked by Tre (Figure 7a). These findings suggest that the transcription of *PpEXPB2* and *PpCNGC1* is positively regulated by the H_2_S‐PpSnRK1α pathway.

To examine whether *PpEXPB2* and *PpCNGC1* transcription is controlled by the interaction of PpSnRK1α with PpLBD16, we analysed the effect of PpSnRK1α on the interactions between PpLBD16 and the target genes. Electrophoretic mobility shift assays (EMSA) showed that PpLBD16‐GST could bind to the biotin‐labelled probes of *PpEXPB2* and *PpCNGC1*, and that co‐incubation with HIS‐PpSnRK1α facilitated this binding, while no binding bands were observed between the probes and GST or HIS‐PpSnRK1α (Figure 7b). In addition, through dual‐luciferase reporter assays, we found that PpLBD16 activated the transcription of *PpEXPB2* and *PpCNGC1*, whereas cotransformation of PpSnRK1α intensified the regulation of PpLBD16 on these two genes (Figure 7c,d). These results suggest that the PpSnRK1α‐PpLBD16 interaction enhances the transcriptional activation of PpLBD16 on the target genes related to LR development.

Silencing of *PpLBD16*, *PpEXPB2* or *PpCNGC1* inhibits peach root growth, reducing LR formation (Wu *et al*., 2024; Figure 7e,j). Here, we further analysed the responses of peach roots silenced with *PpLBD16*, *PpEXPB2* or *PpCNGC1* to exogenous H_2_S (Figure 7e). Consistent with the above results, the application of NaHS significantly increased the expression of *PpEXPB2* and *PpCNGC1* in the *TRV2* line's root and also enhanced the transcription of *PpLBD16* (Figure 7f,g,h). Under NaHS treatment, we observed that there was no significant difference in the number of adventitious roots between these lines (Figure 7i). However, silencing *PpLBD16*, *PpEXPB2* or *PpCNGC1* markedly decreased exogenous H_2_S‐induced LR formation in comparison to the *TRV2* line, which led to a significantly lower number of LRs, particularly the silencing of *PpCNGC1* (Figure 7j). These findings indicate that *PpEXPB2* and *PpCNGC1* are involved in the H_2_S‐PpSnRK1α‐PpLBD16 regulatory pathway for LR development and that *PpCNGC1* may play a key role in H_2_S‐promoted LR formation.

### *PpSnRK1α* , *PpLBD16* and their target genes *PpEXPB2* and *PpCNGC1* are expressed in LR primordia, co‐regulating LR formation

**Figure 8:**
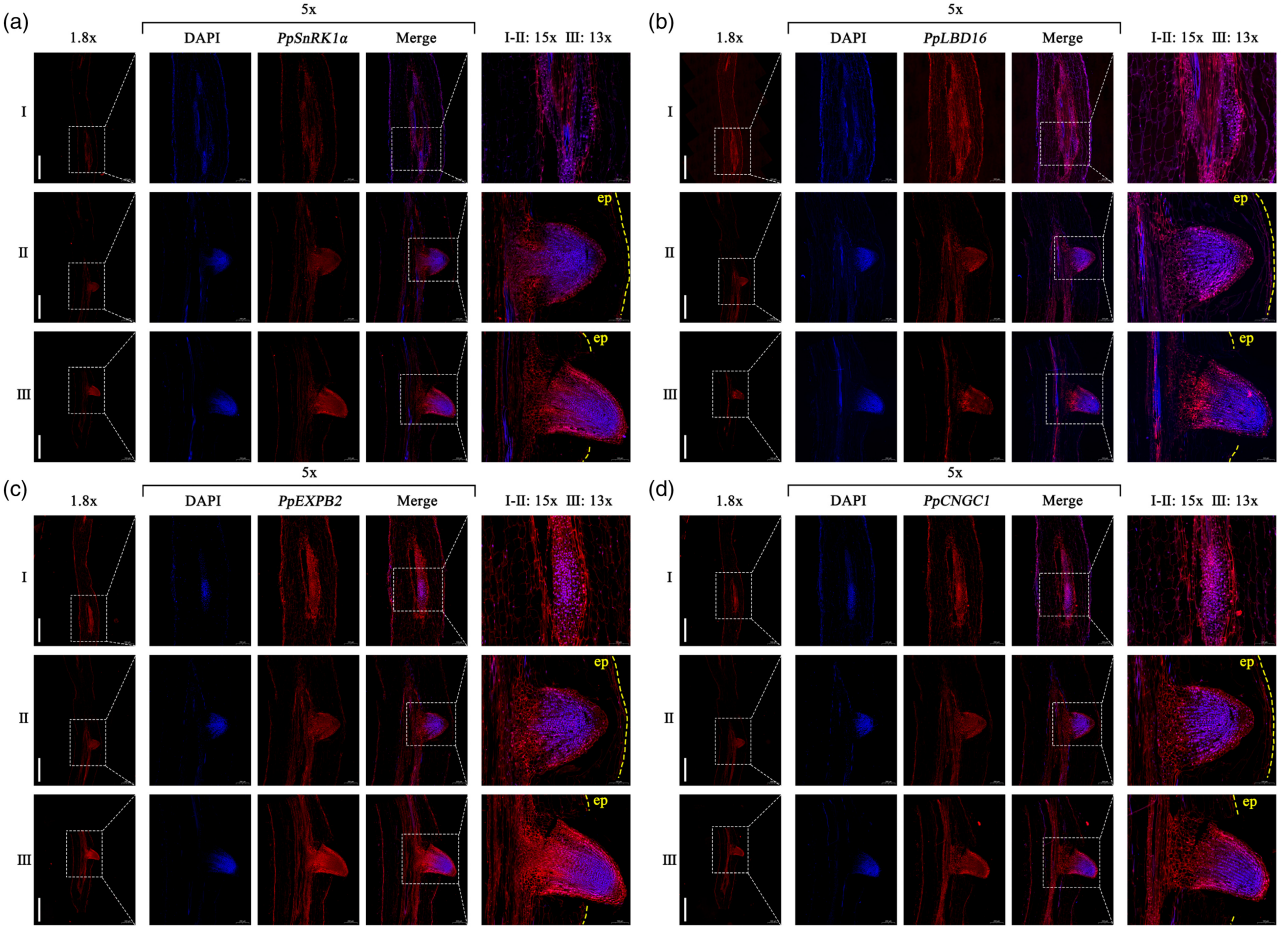
*PpSnRK1α*, *PpLBD16*, *PpEXPB2* and *PpCNGC1* are involved in peach LRP development. (a–d) FISH was used to detect the localisation of *PpSnRK1α* (a), *PpLBD16* (b), *PpEXPB2* (c) and *PpCNGC1* (d) transcripts in peach root tissues, which include the (I) early, (II) middle and (III) emergence stages of LRP development. Root tissue lengthwise sections were hybridized with the gene‐specific antisense probes. The nuclei were stained with DAPI. Ep, epidermis; scale bars, 1000 μm.

Utilising RT‐qPCR, we detected the expression patterns of *PpSnRK1α*, *PpLBD16*, *PpEXPB2* and *PpCNGC1* during peach LR development. We discovered that all of these genes were highly expressed in LR primordia (LRP) development, and their expression (except *PpCNGC1*) decreased after LR emergence (Figure S4), implying a close association between them and the LRP developmental process. In order to further ascertain the specific localisation of their transcripts, we employed fluorescence *in situ* hybridization (FISH) on peach root tissues from three developmental stages of LRP: (I) early, (II) middle and (III) emergence. With the sense probe serving as a control (Figure S5), we observed that these genes were strongly expressed in LRP using the specific antisense probe (Figure 8a–d). This result aligns with the expression pattern assay, suggesting that PpSnRK1α is involved in the regulation of peach LRP development with PpLBD16 and its target genes, *PpEXPB2* and *PpCNGC1*.

Moreover, against the Col‐0 background, we analysed the root phenotypes of *Arabidopsis* plants that overexpressed *PpSnRK1α* and *PpLBD16* independently or together (Figure S6a,b). Compared to Col‐0, overexpressing *PpSnRK1α* had no significant effect on LR number, but severely impeded the elongation of PR, leading to a notable rise in LR density. Overexpressing *PpLBD16* promoted LR formation, significantly increasing the number and density of LRs. When *PpLBD16* was co‐overexpressed with *PpSnRK1α*, the inhibition of *PpSnRK1α* on PR growth disappeared. Compared with the *PpSnRK1α* overexpressing lines, the LR density decreased due to the restoration of PR growth in the co‐expressed lines, and the LR number was further increased compared with the transgenic lines overexpressing *PpLBD16* alone (Figure S6a,c,d). These findings indicate that LR development can be co‐regulated with PpLBD16 and PpSnRK1α.

We also overexpressed *PpLBD16* in the *atsnrk1.1* mutant (*OEPpLBD16/atsnrk1.1*). We found that, compared to the lines overexpressing *PpLBD16* in the Col‐0 background (*OEPpLBD16/*Col‐0), the number and density of LRs in the *OEPpLBD16/atsnrk1.1* lines were not significant changes (Figure S7a–c). However, NaHS treatment caused significant variations between them, and the *OEPpLBD16/atsnrk1.1* had a lower number and density of LRs than the *OEPpLBD16/*Col‐0 (Figure S7b,c), suggesting that the *atsnrk1.1* mutation reduces the sensitivity of the overexpressing plants' LRs to exogenous H_2_S. Under treatment with the H_2_S scavenger HT, the number and density of LRs were suppressed in each overexpression line in either the Col‐0 or *atsnrk1.1* backgrounds, with no significant differences among them (Figure S7b,c), implying that endogenous H_2_S also plays a role in this process. These results likewise confirm that the SnRK1α‐LBD16 pathway can respond to H_2_S to co‐regulate LR formation.

## Discussion

**Figure 9:**
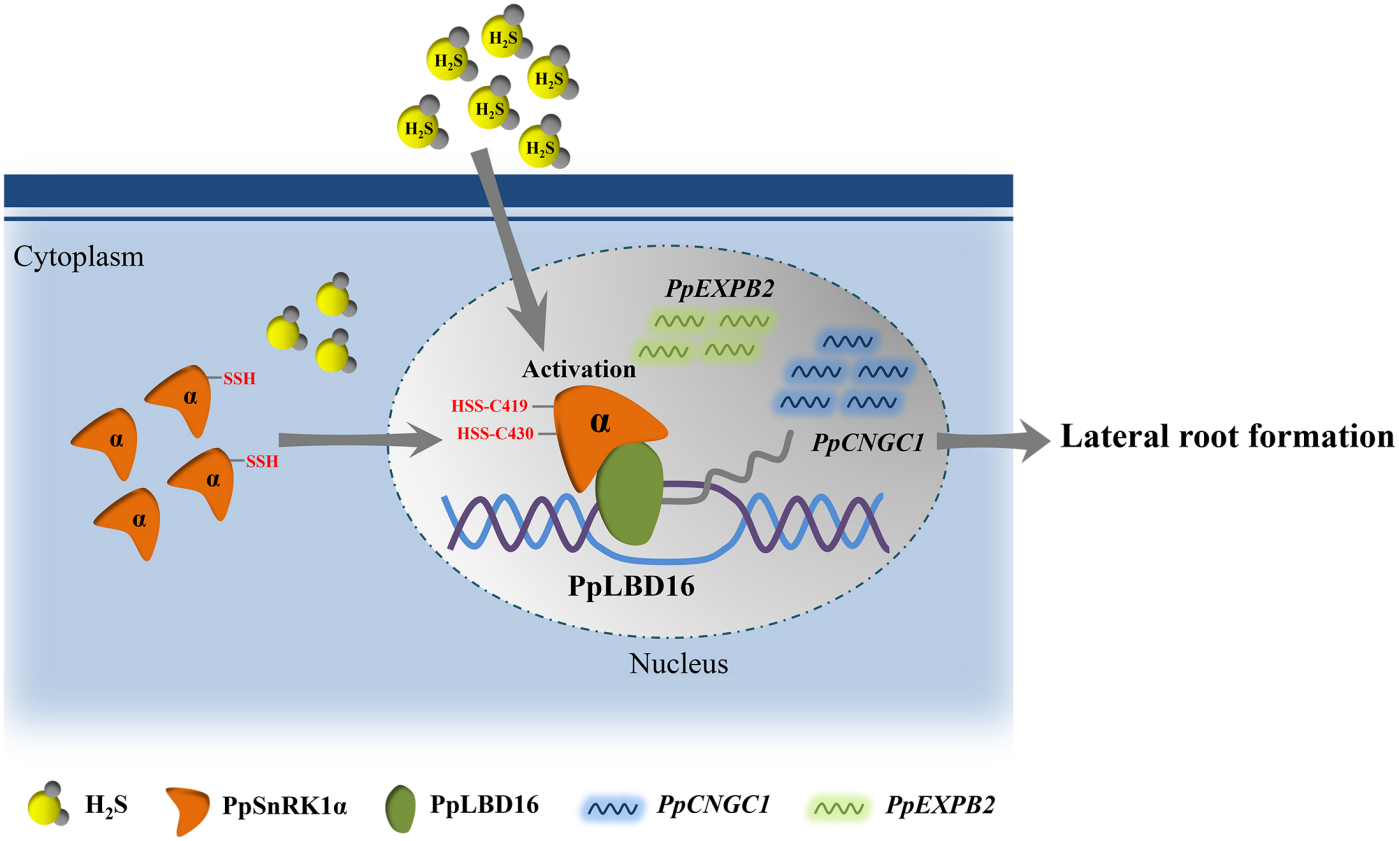
Model for the role of the PpSnRK1α‐PpLBD16 pathway in H_2_S‐induced LR formation. Under the application of exogenous H_2_S, H_2_S activates PpSnRK1α by persulfidating C419 and C430 residues and through its interaction with PpLBD16 in the nucleus, PpSnRK1α increases PpLBD16's transcriptional regulation of the downstream LR development‐associated genes, *PpCNGC1* and *PpEXPB2*, ultimately promoting LR formation. Moreover, the persulfidation by H_2_S leads to the nuclear translocation of PpSnRK1α, enhancing its nuclear localisation, which may strengthen the regulation of the downstream genes.

LR is a critical organ for plants to absorb nutrients. A better understanding of the molecular mechanisms controlling root branching can improve crop growth and thus increase yield. H_2_S has been shown to be involved in the regulation of LR formation (Fang *et al*., 2014; Jia *et al*., 2015; Li *et al*., 2014; Mei *et al*., 2017; Zhang *et al*., 2009a). Here, we propose that the α‐subunit of PpSnRK1 kinase, an energy sensor, mediates H_2_S‐induced LR formation based on persulfidation modification. Through interacting with PpLBD16, a crucial regulatory TF for LR generation, PpSnRK1α increases the transcription of downstream genes related to LR development (Figure 9).

Although we have discovered that both H_2_S and PpSnRK1 regulate the growth of peach LR (Wu *et al*., 2021; Zhang *et al*., 2020), it is unclear whether there is a link between their regulations. In animals, AMPK (homologous to SnRK1) mediates the pharmaceutical effects of H_2_S, which activates AMPK to cure a range of diseases (Chen *et al*., 2016; Kundu *et al*., 2014; Lee *et al*., 2017). In this work, we found that PpSnRK1 activity was enhanced during exogenous H_2_S‐induced peach LR development (Figure 1e,f), which is consistent with studies in animals. After scavenging H_2_S with HT, PpSnRK1 activity was significantly decreased (Figure 1f), indicating that the presence of H_2_S is helpful in preserving PpSnRK1 activity. The SnRK1 catalytic α‐subunit is responsible for the kinase activity, while the regulatory β‐ and γ‐subunits serve as the complex scaffold and energy‐sensing module, respectively (Broeckx *et al*., 2016). The N‐terminal of the α‐subunit has a highly conserved Ser/Thr kinase domain. Phosphorylation of the A‐loop within this domain is essential for kinase activation, and SnRK1α also exhibits independent activity from the regulatory subunits (Ramon *et al*., 2019). Therefore, we speculate that the alteration of PpSnRK1 activity may be due to the effect of H₂S on the α‐subunit.

The redox characteristics of H_2_S may be connected to the altered PpSnRK1 activity. On the one hand, H_2_S reacts with ROS, reducing cellular oxidative damage. On the other hand, H_2_S modifies oxidised Cys by persulfidation, thereby protecting the active Cys residues of target proteins (Corpas and Palma, 2020; Gotor *et al*., 2019). A previous report showed that the redox status of conserved Cys modulates SnRK1α activity, which declines with increasing oxidation (Wurzinger *et al*., 2017), implying that SnRK1α is probably regulated by persulfidation. Based on this speculation, we conducted persulfidation assays on recombinant PpSnRK1α. We detected that NaHS induced the persulfidation of recombinant PpSnRK1α (Figure 2a). Using LC–MS/MS and Cys site mutation analyses, residues C419, C430 and C505 in PpSnRK1α were identified as the persulfidation sites (Figures 2b–d and 3a), and these sites are highly conserved in plants (Figure S1). These evidences demonstrate that PpSnRK1α is a persulfidation target for H_2_S. Unlike other persulfidated proteins (Chen *et al*., 2020; Shen *et al*., 2020), the persulfidation level of PpSnRK1α did not exhibit a NaHS concentration‐dependent increase, which may be caused by the lower concentration of the recombinant PpSnRK1α that saturates the persulfide reaction prematurely.

H_2_S persulfidation acts like GSH S‐glutathionylation to prevent oxidative damage to the protein Cys‐SH groups, and they share similar regulatory mechanisms (Dorion *et al*., 2021; Gotor *et al*., 2019). GSH enhanced SnRK1α activity in *Arabidopsis* (Wurzinger *et al*., 2017). Similarly, we proved that exogenous H_2_S also positively regulates PpSnRK1 activity and persulfidates PpSnRK1α in peach. The persulfidation level and activity of persulfidated target proteins are decreased by the reducing agent DTT (Shen *et al*., 2020). We found that DTT inhibited the persulfidation of PpSnRK1α, as did mutations of residues C419, C430 and C505 in PpSnRK1α (Figures 2a and 3a), and considerably reduced the kinase activity (Figure 3e), suggesting that there is a higher activity of persulfidated PpSnRK1α. In addition, H_2_S‐induced LR formation was inhibited when PpSnRK1 activation was blocked by Tre (Figure 1g–j). Mutations in C419 and C430 residues in PpSnRK1α seriously hindered the H_2_S‐PpSnRK1α pathway from regulating LR production (Figures 4 and 5). These results indicate that H_2_S promotes LR occurrence by persulfidating PpSnRK1α.

SnRK1, acting as an energy sensor and signal integrator, interacts with several TFs to control plant growth and survival through its α‐subunit (Feng *et al*., 2020; Han *et al*., 2020a, 2020b; Mair *et al*., 2015; Pedrotti *et al*., 2018). The TF LBD16, which is downstream of ARF7/19 in the auxin pathway, is crucial for root initiation (Goh *et al*., 2019; Lee *et al*., 2019; Omary *et al*., 2022; Stitz *et al*., 2023). Our earlier findings indicate that PpLBD16 plays a role in H_2_S‐induced peach LR formation (Wu *et al*., 2021). In the present research, we observed an interaction between PpSnRK1α and PpLBD16 in the nucleus (Figure 6). It has been determined that PpLBD16 targets *PpEXPB2* and *PpCNGC1* for controlling LR development (Wu *et al*., 2024). We found that the interaction between PpSnRK1α and PpLBD16 enhanced the binding of PpLBD16 to these two genes and positively regulated their transcription (Figure 7b–d). This mechanism is comparable to the SnRK1α‐bZIP63‐*ARF19* regulatory module during LR initiation (Muralidhara *et al*., 2021). Under non‐stress conditions, there is more nuclear than cytoplasmic localisation of SnRK1α in the root meristematic tissues of *Arabidopsis* (Belda‐Palazón *et al*., 2022). Nuclear SnRK1α facilitates an increase in root meristem size and number of cells. Conversely, the growth of root meristematic tissue is inhibited by cytoplasmic SnRK1α (Belda‐Palazón *et al*., 2022; Ramon *et al*., 2019). In low‐energy stress or acidic environments, SnRK1α undergoes nuclear translocation (Ramon *et al*., 2019; Sun *et al*., 2021a). Since H_2_S is a weak acid and persulfidation modification also leads to nuclear translocation of target proteins (Aroca *et al*., 2017b), we preliminarily analysed the effect of H_2_S on the subcellular localisation of PpSnRK1α. We observed that exogenous H_2_S enhanced the nuclear localisation of PpSnRK1α, while mutations in the persulfidation sites C419 and C430 inhibited this induction (Figure S2a,b). From this, it is reasonable to infer that H_2_S stimulation of PpSnRK1α nuclear translocation may facilitate the interaction between PpSnRK1α and PpLBD16 to promote the transcription of downstream genes.

Upon NaHS treatment, the transcription of *PpCNGC1* and *PpEXPB2* in peach roots was indeed noticeably increased (Figure 7a,g,h), whereas this promotion was inhibited by the application of Tre or DTT (Figure 7a). Tre and DTT blocked the induction of PpSnRK1 activity by exogenous H₂S (Figures 1h and 3e), indicating that H_2_S‐induced PpSnRK1 activity positively regulates *PpEXPB2* and *PpCNGC1* transcription. Through phosphorylation, SnRK1α enhances the regulation of downstream gene expression by TFs (Meng *et al*., 2023; Muralidhara *et al*., 2021). At present, it is unclear whether PpSnRK1α modulates PpLBD16 function through phosphorylation. However, given that PpSnRK1α enhanced PpLBD16 binding to and activation of *PpEXPB2* and *PpCNGC1* (Figure 7b–d), it is most likely that H_2_S‐enhanced PpSnRK1 activity promotes the transcription of these genes through the PpSnRK1α‐PpLBD16 interaction.

*PpCNGC1* transcription was strongly induced under exogenous H_2_S treatment (Figure 7a), suggesting that *PpCNGC1* may be a critical gene regulated by the PpSnRK1α‐PpLBD16 pathway in response to exogenous H_2_S. *CNGCs* encode a class of nonselective cation channels that interact with calmodulin (CaM) to control intracellular Ca^2+^ homeostasis (DeFalco *et al*., 2016; Dietrich *et al*., 2020). Interestingly, H_2_S can modulate Ca^2+^ influx and stimulate LR formation through Ca^2+^/CaM signalling (Li *et al*., 2014). H_2_S‐induced persulfidation was also involved in controlling Ca^2+^ influx in guard cells (Chen *et al*., 2020). These findings point to an overlap in the regulatory networks of H_2_S and CNGCs. Notably, CNGCs, Ca^2+^ and SnRK1α all have a role in regulating the nitrate signalling pathway (Wang *et al*., 2021, 2022). These evidences imply that there is a complex regulatory relationship between H_2_S, SnRK1α and CNGCs. It is noteworthy that NaHS‐induced LR formation was not completely suppressed by the *atsnrk1.1* mutation (Figure 4b,c), suggesting that H_2_S may have additional pathways regulating LR development. When PpSnRK1 activity in peach roots was inhibited with Tre, the activation of *PpCNGC1* transcription by NaHS was also not entirely eliminated. However, the use of DTT decreased *PpCNGC1* expression almost to the control level (Figure 7a). These findings show that PpSnRK1α may not be the only persulfidation target controlling *PpCNGC1*.

All things considered, we proffer some options to guide future research directions. (1) SnRK1 is an important regulator of plant growth and environmental adaptation. It was recently reported that auxin signal reduces SnRK1α activity through the IAA14‐AARF7/19 signalling module to trigger LR formation (Morales‐Herrera *et al*., 2023). However, SnRK1α can be activated by the acute stress of short‐term energy deprivation, which in turn promotes *ARF19* transcription to induce LR development (Muralidhara *et al*., 2021). The findings imply that SnRK1α has a complex regulatory mechanism in regulating LR formation, allowing plants to respond promptly to environmental changes. As a gasotransmitter, H₂S can be produced in plants in response to various stresses (Hilal *et al*., 2023). H₂S may act as a messenger between SnRK1 and environmental signals to promote LR formation by activating SnRK1; however, the mediating role of H_2_S needs to be demonstrated. (2) In *Arabidopsis*, default activation and nuclear translocation of SnRK1α regulate metabolic stress and root development (Ramon *et al*., 2019). As discussed above, based on the chemical properties of H_2_S and its persulfidation of SnRK1α, further experiments are needed to determine whether H₂S‐induced nuclear translocation of SnRK1α leads to an enhancement of the SnRK1α‐LBD16 interaction. Additionally, more research is required to ascertain whether SnRK1α phosphorylates LBD16. (3) It has been proposed that SnRK1 plays a dual regulatory role in plant meristem organisation and that basal SnRK1 activity is required for meristem development (Lopes *et al*., 2024). Nevertheless, the regulatory mechanism is unclear. Is the SnRK1α‐LBD16 module part of the regulatory mechanism of the basal activity? This aspect deserves continued in‐depth research. In conclusion, our current research indicates that PpSnRK1α is a key persulfidation target for H_2_S‐induced peach LR development, and that PpSnRK1α‐PpLBD16‐*PpCNGC1* is a crucial pathway for exogenous H_2_S to control LR formation (Figure 9).

## Experimental procedures

### Plant materials, growth conditions and treatments

Roughly equal‐sized peach (*P. persica*) seeds were placed in damp gauze for germination. After germination, the seeds were transferred to seedling pots with quartz sand as the culture substrate and incubated in the plant growth chamber, which had a 16‐h light/8‐h dark cycle, 24 °C temperature and 65 ± 5% relative humidity. Following one week of growth, peach seedlings were treated with water (control) and 200 μM NaHS, respectively, and successive changes in PpSnRK1 activity or *PpSnRK1α* expression in the roots were determined at 0.5, 2, 4, 8 h, 1 and 5 days after treatment. Peach root phenotypes were analysed by treating with control, 200 μM NaHS, 200 μM NaHS + 100μM HT (or added 80 mM Tre/10 mM DTT) and 100 μM HT (or 10 mM DTT) for 5 days. Samples were collected following the second treatment on day 5 for 4 h to examine PpSnRK1 activity or gene expression. Overexpressed or silenced peach seedlings were treated with water and 200 μM NaHS for 5 days after 21–23 days of transformation, respectively, to analyse root phenotypes.

*Arabidopsis* (*A. thaliana*) seeds were sterilised and sown on 0.5x MS (pH 5.7) solid medium containing 1% (w/v) sucrose and 0.8% (w/v) agar, and vernalised for 3 days at 4 °C. Then, the seeds were moved to a lighted incubator set at 22 °C with a 16‐h light/8‐h dark cycle for horizontal cultivation. Following 2–3 days of seed germination, the seedlings were transferred to 0.5x MS medium with or without 200 μM NaHS or 100 μM HT for vertical culture. After 7 days, the root phenotype was observed, and the number and density of emerging LRs were counted.

### Construction of transgenic materials

*Arabidopsis* accession Columbia (Col‐0) was used as wild‐type background in this study. The *atsnrk1.1* (SALK_127939C) mutant was obtained from the *Arabidopsis* Biological Resource Center (ABRC). *PpSnRK1α* and *PpLBD16* coding sequences (CDSs) were amplified from the cDNA of peach roots. The CDSs of *PpSnRK1α* and persulfidation site‐mutated *PpSnRK1α* (*PpSnRK1α* ^*C419/430/505S*^, using HIS‐PpSnRK1α^C419/430/505S^ vector as a template) were cloned and inserted into the 35S‐driven pRI101‐GFP vector, respectively, to generate overexpression of *PpSnRK1α* (*OEPpSnRK1α/atsnrk1.1*) and persulfidation site‐mutated *PpSnRK1α* (*OEPpSnRK1α* ^*C419/430/505S*^ */atsnrk1.1*) lines in the background of *atsnrk1.1*. In addition, the CDS of *PpLBD16* was also cloned into the pRI101‐GFP vector, and together with the constructed pRI101‐PpSnRK1α, to create individual overexpression lines of *PpSnRK1α* (*Pro35S: PpSnRK1α*) and *PpLBD16* (*Pro35S: PpLBD16*) in the Col‐0 background, respectively. *PpSnRK1α* CDS was inserted into the 35S‐driven pCAMBIA‐1300‐mCherry vector to create co‐overexpression lines of *PpSnRK1α* and *PpLBD16* (*Pro35S: PpSnRK1α & PpLBD16*) in the background of *Pro35S: PpLBD16*. The constructed pRI101‐PpLBD16 vector was also used for overexpression lines in the background of mutant *atsnrk1.1*. Following sequencing validation of each vector, the floral dipping method was used to transform them into *Arabidopsis* using *Agrobacterium tumefaciens* GV3101. All primers and restriction sites used for vector construction are listed in Table S1.

To obtain homozygous transgenic materials, the CDSs of *PpSnRK1α*, *PpSnRK1α* ^*C419*^, *PpSnRK1α* ^*C430S*^ and *PpSnRK1α* ^*C419 430S*^ were inserted into the 35S‐driven pRI101‐GFP vector, respectively. For VIGS, plant‐specific siRNAs were designed with the pssRNAit (Ahmed *et al*., 2020). The effective regions of *PpSnRK1α*, *PpLBD16*, *PpEXPB2* and *PpCNGC1* were recombined into the pTRV2‐GFP vector, respectively. The correctly sequenced overexpression or silencing vectors were transformed into *Agrobacterium rhizogenes* K599 for peach transfection. When peach seedlings had six or seven true leaves, the entire root system was cut out and transfected through the wounds. The transformed peach seedlings were returned to the quartz sand and continued to be cultured for rooting. All recombinant primers and restriction sites are provided in Table S1.

### PpSnRK1 activity assay

SnRK1 activity was assayed according to the previously described method with slight modifications (Zhang *et al*., 2009b). The treated peach roots were washed, cut and mixed. A 0.5 g fresh sample was weighed and ground in 1 mL of pre‐cooled extraction buffer containing 100 mM HEPES (pH 8), 25 mM sodium fluoride, 2 mM tetra‐sodium pyrophosphate, 1 mM benzamidine, 0.5 mM EDTA, 0.5 mM EGTA, 1 mM phenylmethylsulfonyl fluoride, 1 mM protease inhibitor (Sigma‐Aldrich) and phosphatase inhibitors (Roche). The supernatant was collected as peach root protein extract and prepared for use following desalination with the Sephadex G‐25 medium column (GE Healthcare, USA). AMARA (Ala‐Met‐Ala‐Arg‐Ala‐Ala‐Ser‐Ala‐Ala‐ Ala‐Leu‐Ala‐Arg‐Arg‐Arg) peptide was used as a substrate, and SnRK1 activity was measured using a Universal Kinase Activity Kit (R&D Systems, USA) according to the manufacturer's instructions.

### Persulfidation detection of recombinant PpSnRK1α

PpSnRK1α was subjected to a persulfidation assay using a modified biotin‐switch method (Mustafa *et al*., 2009). PpSnRK1α CDS was recombinantly integrated into the pET‐32a‐HIS vector (HIS‐PpSnRK1α). PpSnRK1α^C419/430/505S^ protein expression vectors (HIS‐PpSnRK1α^C419/430/505S^) were constructed utilising the Mut Express MultiS Fast Mutagenesis Kit (Vazyme, China) following the manufacturer's instructions. All primers and restriction sites used are shown in Table S1. The sequencing‐validated vectors were transformed into *Escherichia coli* strain BL21 (DE3) for inducing and purifying recombinant HIS‐PpSnRK1α and HIS‐PpSnRK1α^C419/430/505S^. 50–400 μM NaHS and 10 mM DTT were used to increase and reduce the level of protein persulfidation, respectively. The purified recombinant proteins were incubated with NaHS or DTT for 30 min at 4 °C, and then the NaHS/DTT was removed using a Micro BioSpinP6 column (Bio‐Rad). To block free ‐SH groups, the filtrate of proteins was reacted with 20 mM methyl methanethiosulfonate (MMTS) for 20 min at 50 °C. MMTS was removed through the Micro BioSpinP6 column. To label the persulfidated Cys, the recombinant proteins were treated with 4 mM HDPD‐biotin (ApexBio) for 3 h at 25 °C in the dark. By utilising anti‐biotin antibodies (Abcam) in immunoblotting, biotin‐labelled persulfidated proteins were detected. Coomassie blue staining or immunoblotting with anti‐HIS antibodies (Invitrogen, USA) were used to detect the total proteins.

### LC–MS/MS analysis of persulfidated Cys sites on PpSnRK1α

After 30 min of incubation at 4 °C with 200 μM NaHS, purified recombinant HIS‐PpSnRK1α was separated via nonreducing SDS‐PAGE on 10% (w/v) polyacrylamide gels. With Coomassie Brilliant Blue, the gel was stained. PpSnRK1α‐corresponding gel bands were sliced into roughly 1 mm^3^ cubes, destained with a 50 mM ammonium bicarbonate/acetonitrile (1:1, v/v) solution, and alkylated with 50 mM iodoacetamide (Sigma‐Aldrich) for 1 h at room temperature in the dark. Next, the protein was digested for 16 h at 37 °C using trypsin or chymotrypsin (Promega). The peptides were extracted from the digested sample with 5% (v/v) trifluoroacetic acid‐50% (v/v) acetonitrile. After that, the peptides were desalted and dried. The obtained peptides were subjected to nano LC–MS/MS analysis using an Ultimate 3000 system (Thermo Fisher Scientific, USA) coupled to a Q Exactive™ Hybrid Quadrupole‐Orbitrap™ Mass Spectrometer (Thermo Fisher Scientific, USA) with an ESI nanospray source. The parameters of the analytical column were as follows: 300 μm × 5 mm, packed with Acclaim PepMap RPLC C18, 5 μm particle size, with 100 Å pore size; and 150 μm × 150 mm, packed with Acclaim PepMap RPLC C18, 1.9 μm particle size, with 100 Å pore size. Mobile phase A was 0.1% (v/v) formic acid and 2% (v/v) acetonitrile; mobile phase B was 0.1% (v/v) formic acid and 80% (v/v) acetonitrile; the flow rate was 600 nL/min; and the analysis time was 66 min. The raw MS data were analysed and searched against target protein PpSnRK1α using Byonic software. Oxidation (M, + 15.99 Da), carbamidomethyl (C, + 57.02 Da) and sulfide (C, + 31.97 Da) were searched for dynamic modifications. The precursor ion mass tolerance was 20 ppm, fragment mass tolerance was 0.02 Da, and three missed cleavages were allowed.

### Protein modelling

Peach PpSnRK1α and soybean (*G. max*) GmKIN10 have up to 91.44% amino acid sequence identity. Using SWISS‐MODEL (https://swissmodel.expasy.org/) (Waterhouse *et al*., 2018), the protein structure of GmKIN10 (Protein Data Bank identification: I1N4G7) with a Global Model Quality Estimate value of 0.80 was chosen as a template for three‐dimensional homology modelling of PpSnRK1α. The predicted structure of PpSnRK1α was visualised using PyMOL 4.6 (Schrodinger, LLC) software.

### Yeast two‐hybrid assay

Yeast two‐hybrid experiments were performed according to the manufacturer's manual for the Matchmaker GAL4 Two‐Hybrid System (Clontech). The CDSs of PpSnRK1α and PpLBD16 were cloned into the pGBKT7 bait vector (BD‐PpSnRK1α) and the pGADT7 prey vector (AD‐PpLBD16), respectively. Following sequencing validation, the constructs were cotransformed into the yeast strain Y2HGold in various combinations using the PEG/LiAc method. The yeast cotransformed with pGBKT7‐p53 and pGADT7‐T was used as a positive control, whereas the yeast cotransformed with pGBKT7 or pGADT7 was used as a negative control. After 3 days of cultivation on Trp‐ and Leu‐deficient solid medium (SD/−Trp/−Leu, DDO), the yeasts were transferred to Ade‐, His‐, Trp‐ and Leu‐deficient solid medium (SD/−Ade/‐His/−Trp/−Leu, QDO) with or without 100 ng/mL AbA or 100 μg/mL X‐α‐gal for interaction identifications. All primers and restriction sites involved are listed in Table S1.

### BiFC assay

For BiFC assays, the CDS of PpSnRK1α was inserted into the pSPYCE vector (PpSnRK1α‐YFP^C^), while the CDS of PpLBD16 was inserted into the pSPYNE vector (PpLBD16‐YFP^N^). After the constructs were verified by sequencing, they were transformed into *A. tumefaciens* GV3101. To identify protein–protein interactions, *N. benthamiana* leaves were transiently cotransfected with different combinations of *Agrobacterium*, with the empty vector cotransfection group serving as a negative control. After the transfected plants were cultivated for 2 days, fluorescent signals were visualised using a laser scanning confocal microscope (LSM 880, Zeiss) with a 20x objective. DAPI was used as a nuclear dye. YFP signals were detected using a 514 nm excitation in the 520–585 nm range. Nuclear signals were detected using a 405 nm excitation in the 410–480 nm range. The confocal images were processed and analysed using ZEN software. The primers and restriction sites used are listed in Table S1.

### GST pull‐down assay

PpSnRK1α and PpLBD16 CDSs were recombined into the protein expression vectors pET‐32a‐HIS and pGEX‐4 T‐1‐GST, respectively. The correctly sequenced recombinant vectors were transformed into *E. coli* strain BL21 (DE3) for induction and purification of HIS‐PpSnRK1α and GST‐PpLBD16. The GST used as a control was obtained from the pGEX‐4 T‐1 empty vector. Glutathione beads (Beyotime Biotechnology, China) enriched with GST‐PpLBD16 or GST were mixed with HIS‐PpSnRK1α in pull‐down buffer (50 mM Tris–HCl [pH 7.9], 150 mM NaCl, 1% [v/v] NP‐40 and 1 mM EDTA) and incubated for 6 h on ice in a slow rotation. Partial reaction proteins were collected as input, and then the beads were washed 6–8 times, followed by the addition of 1 x SDS loading buffer for boiling. Using anti‐HiS and anti‐GST antibodies (Invitrogen, USA), immunoblotting was used to identify the proteins. The primers and restriction sites used are presented in Table S1.

### Subcellular localisation analysis

PpSnRK1α subcellular localisation analysis was performed with *OEPpSnRK1α/atsnrk1.1* and *OEPpSnRK1α* ^*C419 430S*^ */atsnrk1.1* transgenic seedlings. The seedlings were cultivated for 5 days on 0.5x MS medium and then transferred to 0.5x MS medium with or without 200 μM NaHS for 2 days. Root apical meristems of the seedlings were used to detect GFP signals. DAPI was used as a nuclear dye. The GFP fusion protein was localised using a laser scanning confocal microscope (LSM 880, Zeiss) with a 20x lens. The GFP signal was detected with a 488 nm excitation in the 480–550 nm range. The DAPI signal was detected with a 405 nm excitation in the 410–480 nm range. Images were processed using ZEN software. The mean GFP fluorescence intensity in the nucleus and cytoplasm was quantified using ImageJ software.

### EMSA

With GST and HIS serving as controls, EMSA was carried out using purified HIS‐PpSnRK1α and GST‐PpLBD16 recombinant proteins. Biotin‐labelled promoter probes for *PpEXPB2* and *PpCNGC1* were prepared utilising DNA annealing buffer (Beyotime Biotechnology, China). The primers are shown in Table S1. Binding analysis was conducted using the LightShift^®^ Chemiluminescent kit (Thermo Scientific, USA) according to the manufacturer's instructions. For the same target gene, ensure that the concentration of HIS‐PpSnRK1α or GST‐PpLBD16 added in each reaction was consistent. The reactions were incubated for 30 min at room temperature in the dark and then immunodetected using 6% (v/v) polyacrylamide gels.

### Dual‐luciferase assay

The CDSs of PpSnRK1α and PpLBD16 were recombined into the pGreenII62‐SK vector, respectively. The promoter sequences of *PpEXPB2* (−1663 bp to −1443 bp distant from the transcription start site) and *PpCNGC1* (−42 bp to 155 bp) were recombined into the pGreenII0800‐LUC vector, respectively. Sequencing‐correct vectors were transformed into *A. tumefaciens* GV3101 and injected into the *N. benthamiana* leaf in different areas based on various combinations (see Figure 7c). Following 2 days of growth for transfected plants, 1 mM D‐Luciferin sodium salt solution was applied to the abaxial surface of the leaves. Luciferase signals were then observed and processed using an IVIS LuminaII In‐Vivo Imaging Device (Xenogen, USA). The primers and restriction sites used are listed in Table S1.

### RT‐qPCR analysis

Total RNA was isolated from the root samples using the RNAprep Pure Plant Kit (TIANGEN, China) and was reverse transcribed to first‐strand cDNA using the PrimeScript™ RT Kit (Takara, Japan). qPCR was performed on a QuantStudio^®^ 3 real‐time PCR instrument (Thermo Fisher Scientific, USA) utilising the SYBR^®^ Premix Ex Taq™ (Takara, Japan). For each sample, three biological replicates as well as three technical replicates were carried out. All reagents were used following the manufacturer's protocol. Reference genes for peach and *Arabidopsis* were *PpActin7* and *AtActin7*, respectively. The 2^−ΔΔCT^ method was used to analyse the relative expression of the genes. Primers used for RT‐qPCR are shown in Table S1.

### FISH

RNA *in situ* hybridization was performed as previously described with slight modifications (Huang *et al*., 2019; Liu *et al*., 2020). The specific probes for *PpSnRK1α*, *PpLBD16*, *PpEXPB2* and *PpCNGC1* labelled with digoxygenin were designed and synthesised by Sangon Biotech (China) and have been listed in Table S1. Root tissues were sampled from 8‐day‐old peach seedlings. After fixing, dehydrating and embedding in wax, paraffin blocks of the samples were sliced 6 μm thick by a slicing machine. Completing dewaxing, tissue sections were digested with proteinase K (20 μg/mL) for 1 h at 37 °C, then prehybridised for 1 h at 37 °C and incubated with RNA probes at 37 °C overnight. Following washing, tissue slices were hybridized with biotin‐conjugated mouse anti‐digoxin antibody (Jackson, USA) and treated with horseradish peroxidase–labelled streptavidin (Jackson, USA). Next, a Tyramide Lumo Kit (MSBIO, China) was used to amplify the signal. After staining the cell nuclei with DAPI, the sections were viewed and photographed under a fluorescent microscope (Eclipse CI, Nikon).

### Statistical analysis

Statistical analyses of the data were performed using GraphPad Prism 7.0 software. One/two‐way ANOVA followed by Tukey's multiple comparison was used to evaluate statistical significance between different groups, denoted as different letters or asterisks (****P < 0.0001, ***P < 0.001, **P < 0.01, *P < 0.05, and ns = not significant). Data are presented as mean, mean ± SD (error bar) or boxplots.

## Conflict of interest

The authors have declared no conflict of interest.

## Author contributions

X.L.W., F.T.P. and Y.S.X. conceived and designed the research; F.T.P. and Y.S.X. supervised the research; X.L.W. and A.Q.D. performed the experiments. X.L.W., A.Q.D., J.H.L. and Z.W. analysed the data; X.L.W., Y.S.X. and F.T.P. wrote the paper.

## Supporting information


**Figure S1** Protein sequence alignment of the SnRK1α in *Prunus persica* (PpSnRK1α), *Arabidopsis thaliana* (AtSnRK1.1) and *Glycine max* (GmKIN10).
**Figure S2** H_2_S enhances nuclear localisation of PpSnRK1α.
**Figure S3** *PpSBT1.7* (a) and *PpPPO* (b) relative expression in the roots of control, NaHS, HT, NaHS + Tre and NaHS + DTT‐treated peach seedlings was determined by RT‐qPCR.
**Figure S4** Expression patterns of *PpSnRK1α*, *PpLBD16*, *PpEXPB2* and *PpCNGC1* during LR development were detected by RT‐qPCR.
**Figure S5** Peach root tissues hybridised with sense probes for *PpSnRK1α*, *PpLBD16*, *PpEXPB2* and *PpCNGC1* were observed by FISH.
**Figure S6** LR development is co‐regulated by PpSnRK1α and PpLBD16.
**Figure S7** Mutation *atsnrk1.1* attenuates the sensitivity of overexpressed *PpLBD16* transgenic *Arabidopsis* LR to exogenous H_2_S.
**Table S1** All primers and probes used in this study.

## Data Availability

All data generated are available in the article or extended data. Sequence data from this article can be found in the National Center for Biotechnology Information (NCBI) database or the *Arabidopsis* Information Resource database (TAIR) under the following accession numbers: *PpSnRK1α* (LOC18781882); *PpLBD16* (LOC18768840); *PpEXPB2* (LOC18787730); *PpCNGC1* (LOC18786740); *PpSBT1.7* (LOC18793338); *PpPPO* (LOC18778665); *PpActin7* (LOC18779708); *AtSnRK1.1* (AT3G01090); *AtActin7* (AT5G09810); and *GmKIN10* (LOC100796687).
