# Supplementary material for: Hydrogen sulfide promotes lateral root formation in peach through persulfidation of SnRK1α kinase
Source: Plant Biotechnol J. 2025 Jul 4;23(10):4395–411. doi: 10.1111/pbi.70245 (PMC12483961; doi:10.1111/pbi.70245)
Supplement: Supplementary file 1 — Figure S1 Protein sequence alignment of the SnRK1α in Prunus persica (PpSnRK1α), Arabidopsis thaliana (AtSnRK1.1) and Glycine max (GmKIN10). Figure S2 H2S enhances nuclear localisation of PpSnRK1α. Figure S3 PpSBT1.7 (a) and PpPPO (b) relative expression in the roots of control, NaHS, HT, NaHS + Tre and NaHS + DTT‐treated peach seedlings was determined by RT‐qPCR. Figure S4 Expression patterns of PpSnRK1α, PpLBD16, PpEXPB2 and PpCNGC1 during LR development were detected by RT‐qPCR. Figure S5 Peach root tissues hybridised with sense probes for PpSnRK1α, PpLBD16, PpEXPB2 and PpCNGC1 were observed by FISH. Figure S6 LR development is co‐regulated by PpSnRK1α and PpLBD16. Figure S7 Mutation atsnrk1.1 attenuates the sensitivity of overexpressed PpLBD16 transgenic Arabidopsis LR to exogenous H2S. Table S1 All primers and probes used in this study. [file PBI-23-4395-s001.pdf]

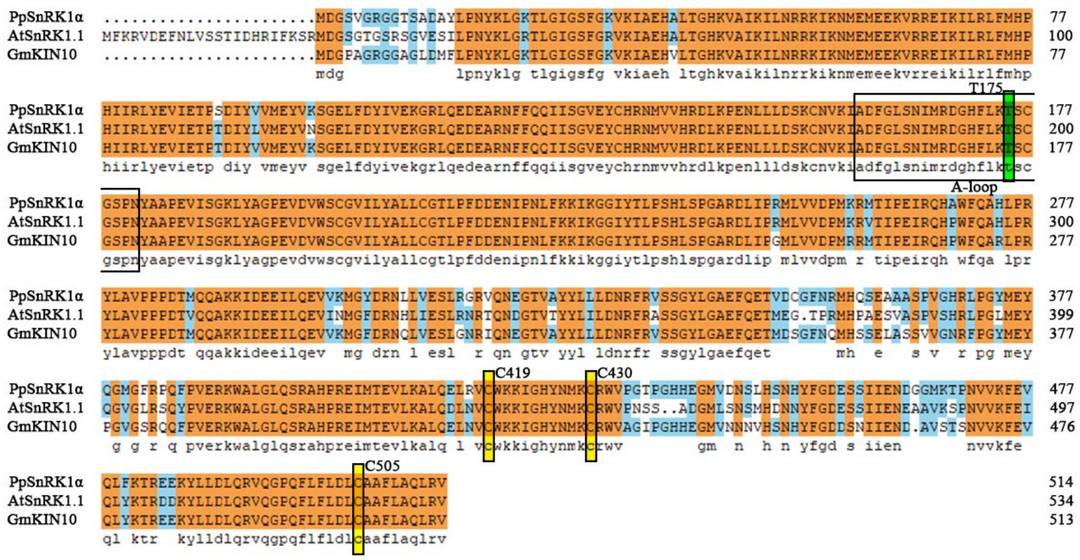

Figure S1 Protein sequence alignment of the SnRK1α in *Prunus persica* (PpSnRK1α), *Arabidopsis thaliana* (AtSnRK1.1), and *Glycine max* (GmKIN10). The A-loop is the activation loop; T175 is the critical phosphorylation site for the kinase activity; C419, C430, and C505 are the persulfidation sites of PpSnRK1α.

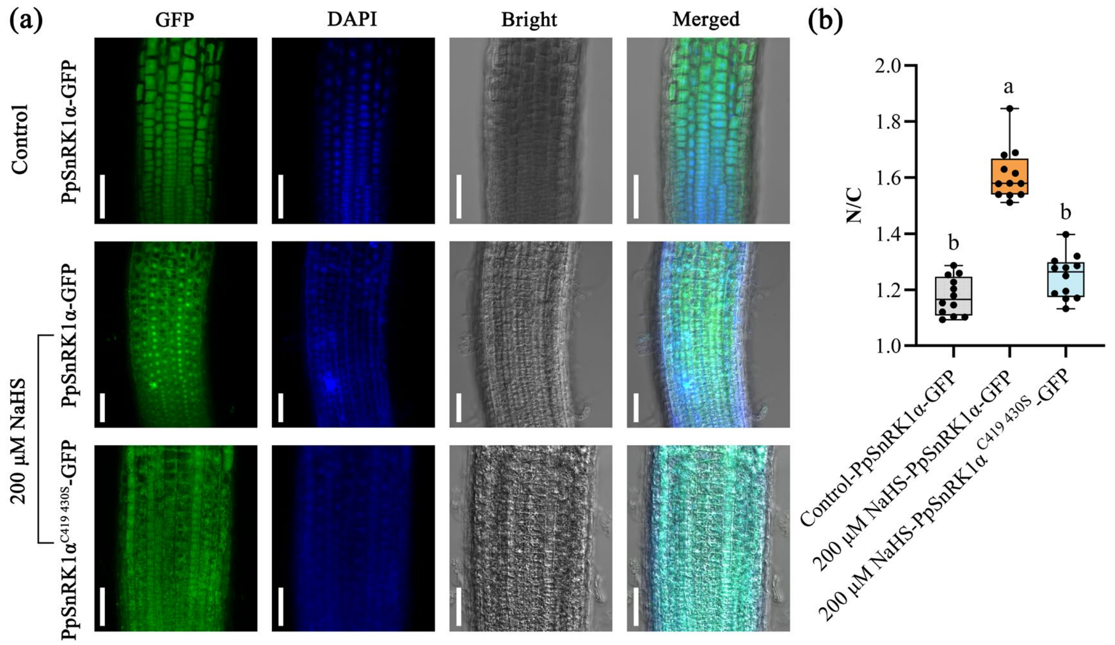

Figure S2 H_2S enhances nuclear localization of PpSnRK1α. (a) Effect of exogenous H_2S on the subcellular localization of PpSnRK1α. With or without 200 μM NaHS treatment (2 d), root apical meristems of 7-day-old *OEPpSnRK1α/atsnrk1.1* and *OEPpSnRK1α$^{C419\ 430S}$/atsnrk1.1 Arabidopsis* seedlings were used for observation. The nuclei were stained with DAPI. Scale bars, 50 μm. (b) Quantitation of the ratio of mean nuclear and mean cytoplasmic GFP fluorescence intensity (N/C) in PpSnRK1α-GFP-expressing cells and PpSnRK1α$^{C419\ 430S}$-GFP-expressing cells with or without 200 μM NaHS treatment. The nuclear and cytoplasmic mean GFP fluorescence intensity of the cells was measured using ImageJ software and used to calculate the N/C ratio, n = 12. Center line = median; whiskers = highest and lowest values; box limits = upper and lower quartiles; points = single measures. Statistical analyses were performed with one-way ANOVA. Different letters denote statistically significant differences ($P < 0.05$).

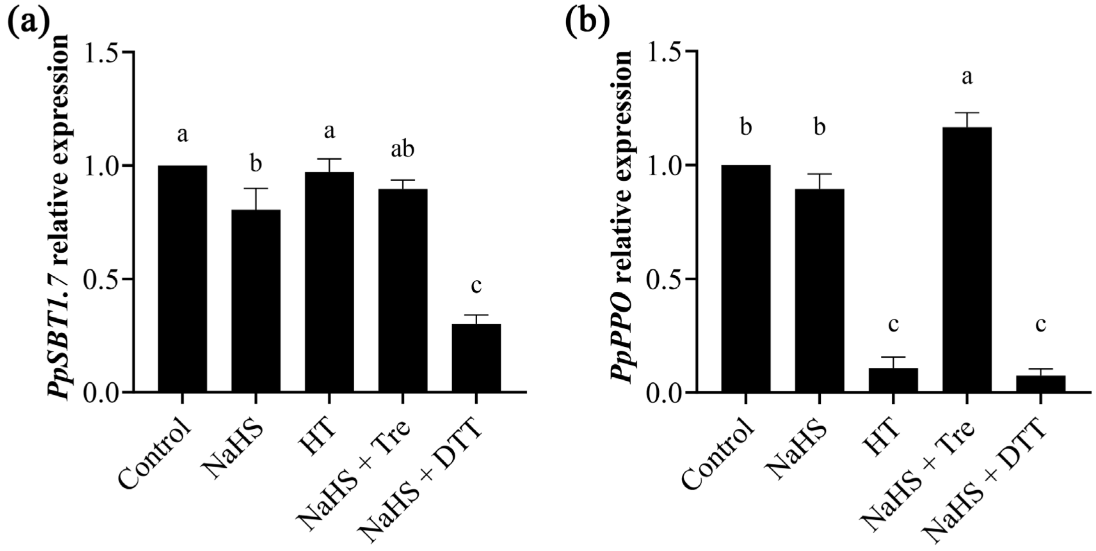

Figure S3 *PpSBT1.7* (a) and *PpPPO* (b) relative expression in the roots of control, NaHS, HT, NaHS + Tre, and NaHS + DTT-treated peach seedlings was determined by RT-qPCR. One further treatment was applied to peach seedlings on the 5th day following the initial one. Samples for RT-qPCR analysis were taken at 4 h after the 2nd treatment. The relative expression of the genes for each treatment was calculated based on the comparison with the level of the control group (1.00). Data are means with SDs of three biological replicates. Statistical analyses were performed with one-way ANOVA. Different letters denote statistically significant differences (P < 0.05).

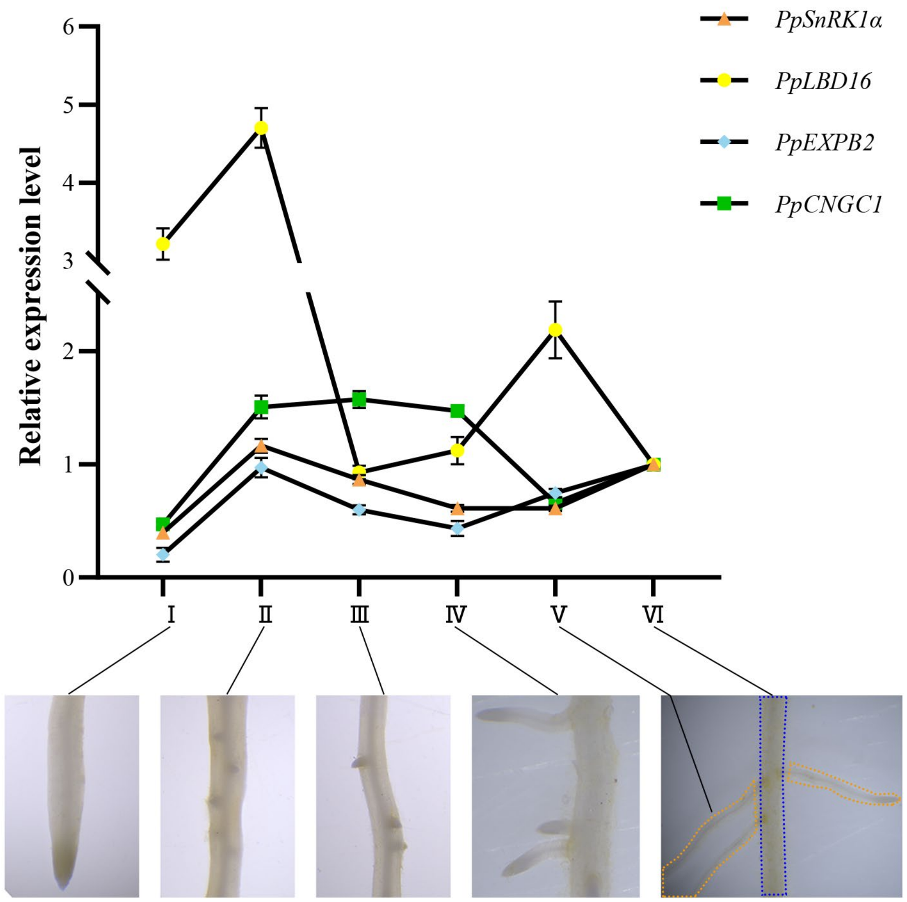

Figure S4 Expression patterns of *PpSnRK1α*, *PpLBD16*, *PpEXPB2*, and *PpCNGC1* during LR development were detected by RT-qPCR. To investigate the expression patterns of these genes during LR development, the root of the 8-d-old peach seedling was sampled in segments under a stereoscopic microscope. The root was divided into six sections: (I) root tip, (II) LRP development, (III) LR emergence, (IV) LR elongation, (V) LR developing maturation, and (VI) primary root region corresponding to V, which represent various LR developmental stages. Data are mean with SD of three biological replicates.

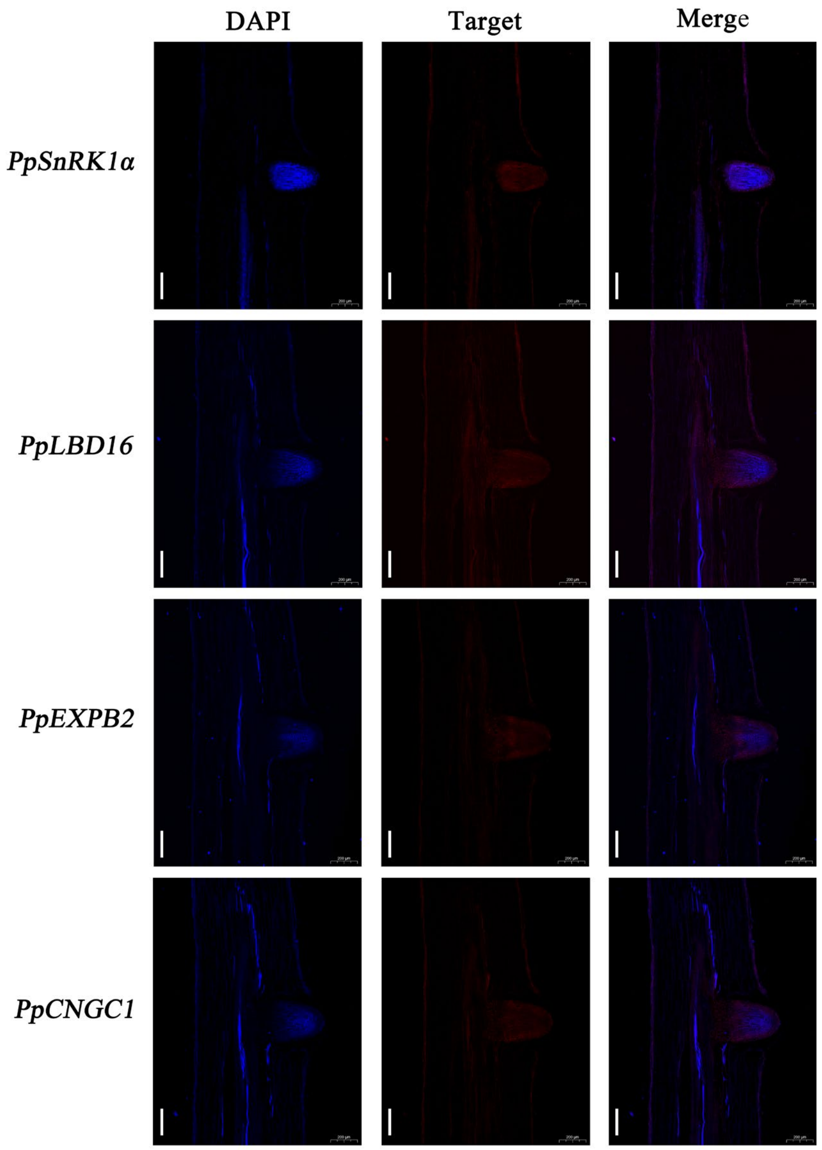

Figure S5 Peach root tissues hybridized with sense probes for *PpSnRK1α*, *PpLBD16*, *PpEXPB2*, and *PpCNGC1* were observed by FISH. Peach root tissue of LRP emergence stage (III) was used. Scale bars, 200 μm.

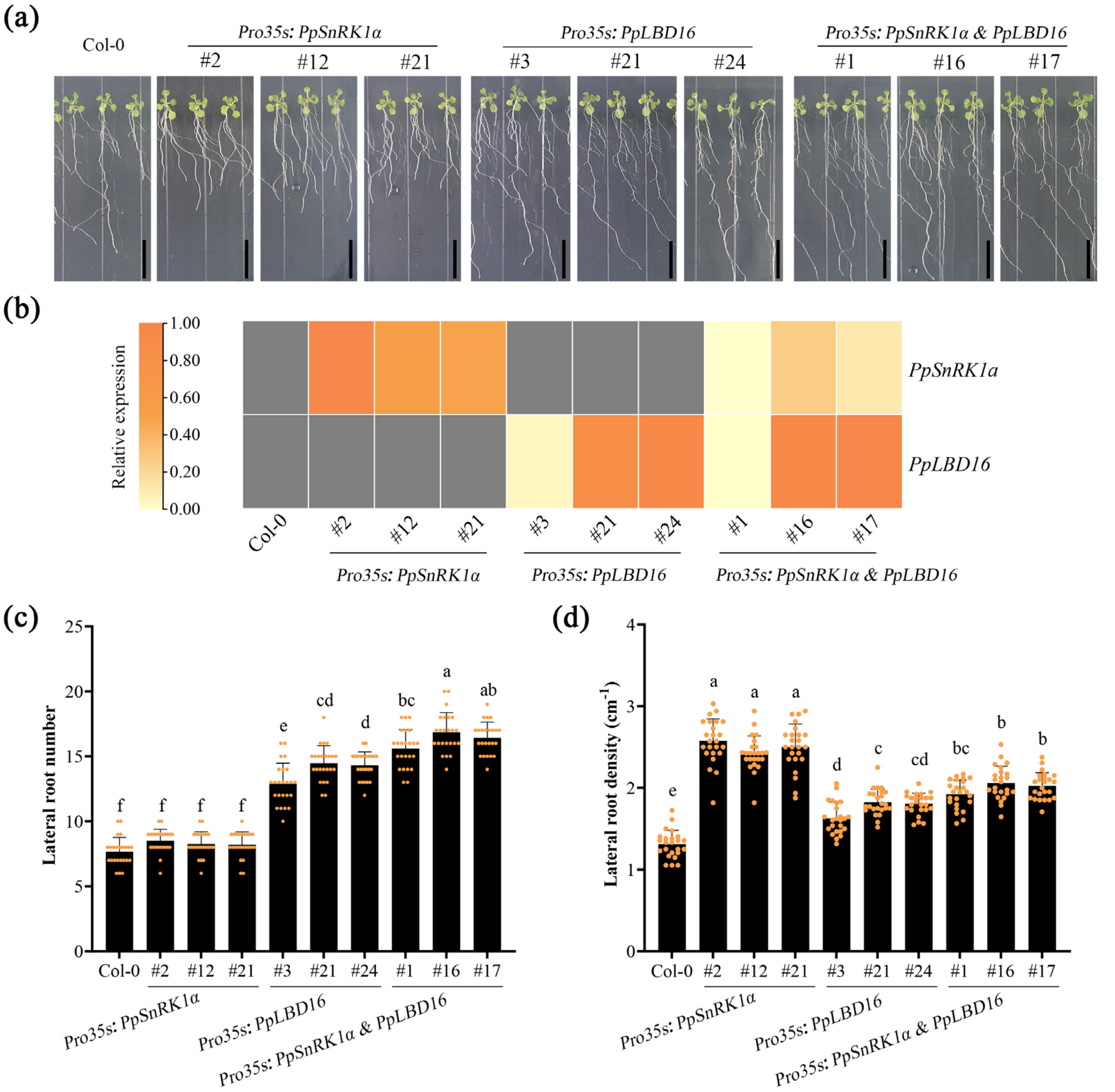

Figure S6 LR development is co-regulated by PpSnRK1α and PpLBD16.

(a) Root phenotypes of 10-day-old transgenic *Arabidopsis* overexpressing *PpSnRK1α* and *PpLBD16* alone (*Pro35s: PpSnRK1α* and *Pro35s: PpLBD16*) or together (*Pro35s: PpSnRK1α* & *PpLBD16*) in a Col-0 background. Scale bars, 1.5 cm.

(b) Relative expression of *PpSnRK1α* and *PpLBD16* in the *Arabidopsis* roots of the transgenic lines in (a). The heatmap was standardized by row Z-score. Grey color represents no expression of the gene.

(c and d) LR number (c) and density (d) of *Arabidopsis* in (a), n = 24. Data are means with SDs of biological replicates (n). Statistical analyses were performed with one-way ANOVA. Different letters denote statistically significant differences ($P < 0.05$).

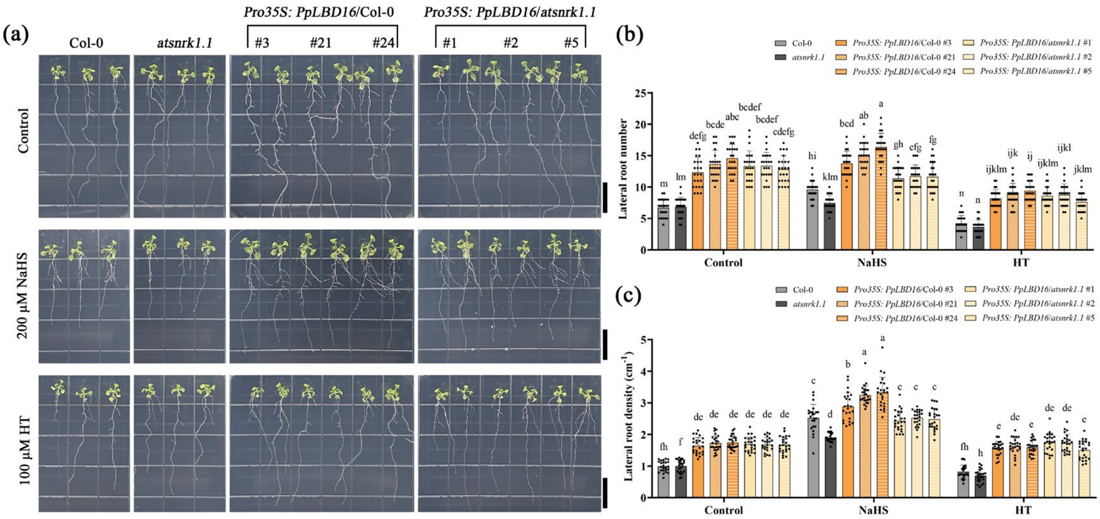

Figure S7 Mutation *atsnrk1.1* attenuates the sensitivity of overexpressed *PpLBD16* transgenic *Arabidopsis* LR to exogenous H_2S.

(a) Root phenotypes of 9-day-old transgenic *Arabidopsis* overexpressing *PpLBD16* (*Pro35s: PpLBD16*) in the Col-0 background or *atsnrk1.1* background. Seedlings were grown on 0.5x MS medium with or without 200 μM NaHS or 100 μM HT. Scale bars, 1.5 cm.

(b and c) LR number (b) and density (c) of *Arabidopsis* in (a), n = 24. Data are means with SDs of biological replicates (n). Statistical analyses were performed with two-way ANOVA. Different letters denote statistically significant differences (P < 0.05).

Table S1 All primers and probes used in this study.

Prime name/ restriction sites	Prime sequence (5'- 3')
**Primers used for RT-qPCR**	
PpActin7-F	GTTATTCTTCATCGGCGTCTTCG
PpActin7-R	CTTCACCATTCCAGTTCCATTGTC
PpSnRK1α-F	CCTGAAGTGGATGTGTGGAGTTG
PpSnRK1α-R	TCAAGTCTCTTGCACCAGGTGAC
PpEXPB2-F	AGCAAAGTGGTGGAGACTCG
PpEXPB2-R	GTTTGCTACAAGGGCTTGGC
PpCNGC1-F	GGCGCATAGACACAGGATGA
PpCNGC1-R	TCATGCAAGCCTTTGGGGAT
PpSBT1.7-F	GGCATCAGAGAAACTCGGCA
PpSBT1.7-R	CTTCCTCACACTGCCCTCTC
PpPPO-F	TCCCATCTTCTTCGCACACC
PpPPO-R	AGAAACGAGCTGTCAAGCCA
PpLBD16-F	GGAACATAGAGAGCACTCAGTGGC
PpLBD16-R	GCTTGGTATGAGAAGTCATCTCTGC
AtActin7-F	TCGGAGCTGAGAGATTCCGT
AtActin7-R	TGGAACCACCACTGAGAACG
**Primers used for recombinant protein**	
pET-32a-PpSnRK1α-F/BamHI	gccatggctgatatcggatccATGGATGGATCGGTTGGCC
pET-32a-PpSnRK1α-R/SalI	tgcggccgcaagcttgtcgacAAGGACCCGAAGTTGTGCAA
PpSnRK1α-C419S-F	GCGGGTTTcTTGGAAGAAGATAGGGCACTACAA
PpSnRK1α-C419S-R	TCTTCCAAgAAACCCGCAATTCTTGTAAAGC
PpSnRK1α-C430S-F	CATGAAGTcTAGGTGGGTTCCTGGAACTCC
PpSnRK1α-C430S-R	CCCACCTAgACTTCATGTTGTAGTGCCCTATCTTC
PpSnRK1α-C505S-F	GGATCTTTcTGCTGCTTTCCTTGCACAACT
PpSnRK1α-C505S-R	AAGCAGCAgAAAGATCCAAGAAGAGAAACTGGG
PpSnRK1α-C419 430S-F	GGAAGAAGATAGGGCACTACAACATGAAGTcTAGGTGGGTTCCTGGAACTCC
PpSnRK1α-C419 430S-R	AGTGCCCTATCTTCTTCCAAgAAACCCGCAATTCTTGTAAAGC
pGEX-4T-1-PpLBD16-F/BamHI	gatctggttccgcgtggatccATGGCTTCTGGGGCTGGC
pGEX-4T-1-PpLBD16-R/SalI	gatgcggccgctcgagtcgacGTTTCTCATCATTCTGAGGGCC
**Primers used for transgenic *Arabidopsis* lines**	
pRI101-PpSnRK1α-F/SalI	ttgatacatatgcccgtcgacATGGATGGATCGGTTGGCC
pRI101-PpSnRK1α-R/BamHI	gttgattcagaattcggatccAAGGACCCGAAGTTGTGCAA
pRI101-PpLBD16-F/Nde I	cgccatatgATGGCTTCTGGGGCTGG
pRI101-PpLBD16-R/EcoRI	ccggaattcGTTTCTCATCATTCTGAGGGCC
pCAMBIA1300-PpSnRK1α-F/SalI	gcttcgaattctgcagtcgacATGGATGGATCGGTTGGCC
pCAMBIA1300-PpSnRK1α-R/BamHI	ccttgctcaccatcaggatccAAAGGACCCGAAGTTGTGCA
**Primers used for transgenic peach lines**	
pRI101-PpSnRK1α-F/SalI	ttgatacatatgcccgtcgacATGGATGGATCGGTTGGCC
pRI101-PpSnRK1α-R/BamHI	gttgattcagaattcggatccAAGGACCCGAAGTTGTGCAA
pTRV2-PpSnRK1α-F/EcoRI	gtgagtaaggttaccgaattcATCAAAATACTGAGACTGTTTATGCATC

pTRV2-PpSnRK1α-R/BamHI	cgtgagctcggtaccggatccTGACAATACTCTACGCCCGAGA
pTRV2-PpLBD16-F/EcoRI	gtgagtaaggttaccgaattcAGCAATGTGTCCAAATTGTTGTTG
pTRV2-PpLBD16-R/BamHI	cgtgagctcggtaccggatccTTCATAGCAATATCACTGCAGTTGTG
pTRV2-PpEXPB2-F/EcoRI	gtgagtaaggttaccgaattcACGGAATGCTGGAGTCCTGC
pTRV2-PpEXPB2-R/BamHI	cgtgagctcggtaccggatccGGGATCGATAAGTCTGGCCG
pTRV2-PpCNGC1-F/EcoRI	gtgagtaaggttaccgaattcATCATGGAGAAGGTACAACAAGAAGT
pTRV2-PpCNGC1-R/BamHI	cgtgagctcggtaccggatccATACTTTTCCTGCACGTCGAGG
**Primers used for yeast two-hybrid assay**	
pGBKT7-PpSnRK1α-F/EcoRI	atggccatggaggccgaattcATGGATGGATCGGTTGGCC
pGBKT7-PpSnRK1α-R/SalI	atgcggccgctgcaggtcgacTTAAAGGACCCGAAGTTGTGCA
pGADT7-PpLBD16-F/EcoRI	gccatggaggccagtgaattcATGGCTTCTGGGGCTGGC
pGADT7-PpLBD16-R/BamHI	cagctcgagctcgatggatccGTTTCTCATCATTCTGAGGGCC
**Primers used for BIFC**	
pSPYCE-PpSnRK1α-F/BamHI	tggcgcgccactagtggatccATGGATGGATCGGTTGGCC
pSPYCE-PpSnRK1α-R/SalI	agcggtaccctcgaggtcgacAAGGACCCGAAGTTGTGCAA
pSPYNE-PpLBD16-F/BamHI	tggcgcgccactagtggatccATGGCTTCTGGGGCTGGC
pSPYNE-PpLBD16-R/SalI	agcggtaccctcgaggtcgacGTTTCTCATCATTCTGAGGGCC
**Primers used for EMSA**	
3'Biotin-proPpEXPB2-F	TATAATAATAAAGAAAAGAGGAAAAAAGACTTGTA
3'Biotin-proPpEXPB2-R	TACAAGTCTTTTTTCCTCTTTTCTTTATTATTATA
3'Biotin-proPpCNGC1-F	AGAGTTTCCCAATTTTACACTAATTTGTTGTTTTTATTTTTTATTTTCTACA
3'Biotin-proPpCNGC1-R	TGTAGAAAATAAAAAATAAAAACAACAAATTAGTGTAAAATTGGGAAACTCT
**Primers used for dual-luciferase assay**	
pGreenII62-SK-PpSnRK1α-F/BamHI	cgctctagaactagtggatccATGGATGGATCGGTTGGCC
pGreenII62-SK- PpSnRK1α-R/EcoRI	gataagcttgatatcgaattcTTAAAGGACCCGAAGTTGTGCA
pGreenII62-SK-PpLBD16-F/BamHI	cgctctagaactagtggatccATGGCTTCTGGGGCTGGC
pGreenII62-SK-PpLBD16-R/EcoRI	gataagcttgatatcgaattcTCAGTTTCTCATCATTCTGAGGGC
pGreenII0800-proPpEXPB2-F/SalI	gggccccccctcgaggtcgacTATAATAATAAAGAAAAGAGGAAAAAAGACTTG
pGreenII0800-proPpEXPB2-R/BamHI	cgctctagaactagtggatccATTTCGTACCTAGGTATTGTGCATTT
pGreenII0800-proPpCNGC1-F/SalI	gggccccccctcgaggtcgacATCTGCAGTGACTTGAGATATATATGTTG
pGreenII0800-proPpCNGC1-R/BamHI	cgctctagaactagtggatccGCCATTGTTGCCTCTGAATTTG
**Probes used for FISH**	
5'Dig-PpSnRK1α-3'Dig-antisense	CCGCAACUCCACACAUCCACUUC
5'Dig-PpSnRK1α-3'Dig-sense	GAAGUGGAUGUGUGGAGUUGCGG
5'Dig-PpLBD16-3'Dig-antisense	ACAGUCAGAGGCACACUUUC
5'Dig-PpLBD16-3'Dig-sense	GAAAGUGUGCCUCUGACUGU
5'Dig-PpEXPB2-3'Dig-antisense	ACCGUCGGGGUUUCCAUACCAAGU
5'Dig-PpEXPB2-3'Dig-sense	ACUUGGUAUGGAAACCCCGACGGU
5'Dig-PpCNGC1-3'Dig-antisense	UCGUCGUAGACAUUAUCGCUCG
5'Dig-PpCNGC1-3'Dig-sense	CGAGCGAUAAUGUCUACGACGA
